# Perceptual constraints on colours induce the universality of linguistic colour categorisation

**DOI:** 10.1038/s41598-019-44202-6

**Published:** 2019-05-22

**Authors:** Tao Gong, Hangxian Gao, Zhen Wang, Lan Shuai

**Affiliations:** 1 Centre for Linguistics and Applied Linguistics, Guangdong University of Foreign Studies, Guangzhou, China; 2 Educational Testing Service, Princeton, NJ USA; 3 School of Materials and Chemical Technology, Tokyo Institute of Technology, Tokyo, Japan; 4 School of Cyberspace, Hangzhou Dianzi University, Hangzhou, China

**Keywords:** Evolution of language, Human behaviour

## Abstract

The universal linguistic colour categorisation pattern as evident in the World Colour Survey (WCS) has been a principal focus of investigations on the relationship between language and cognition, yet most existing studies have failed to clarify whether this universality resulted primarily from individual perceptual constraints and/or socio-cultural transmissions. This paper designed an agent-based, unsupervised learning model to address the relative importance of these two aspects to linguistic colour categorisation. By directly comparing with the empirical data in the WCS, our study demonstrated that: the physical colour stimuli that reflect human perceptual constraints on colours trigger a categorisation pattern quantitatively resembling the WCS data, the randomised stimuli that distort such constraints lead to distinct categorisation patterns, and the processes of linguistic categorisation in both cases follow similar dynamics. These results reveal how perceptual and socio-cultural factors interact with each other to trigger linguistic universality, and serve as decisive evidence that human perceptual constraints induce the universality in linguistic categorisation, yet socio-cultural transmissions, though imperative, play an auxiliary role of transcribing perceptual constraints into common linguistic categories with slight variations.

## Introduction

Humans make use of their fine-grained perceptual systems to discriminate a wide range of apparently continuous sensory stimuli such as colours, whereas different languages usually organise these stimuli discretely into a relatively small set of descriptive categories. Linguistic categorisation of colours, as an individual behaviour and a community conventionalisation process, is subject to constraints from both individual perception and socio-cultural transmissions. This dual nature makes it an appropriate test bed on evolutionary relations between language and perception^1,2^.

An influential theory on this issue holds that the linguistic colour categorisation pattern is determined predominately by primitives in human perceptual system (and ecological environment)^3–5^. Some scholars even claim the innateness of colour categories in human genomes^6^. Another hypothesis stresses that the universality mainly results from boundary demarcation imposed by individual languages and socio-cultural factors^7–9^. Recently, a third view suggests that both individual perception and socio-cultural transmissions are equally important and indispensable to linguistic categorisation^10–13^. Clarifying these competing views requires a detailed evaluation of the relative importance of perceptual constraints and socio-cultural factors during linguistic colour categorisation.

The World Colour Survey (WCS) dataset provides an empirical foundation for such evaluation (see Supplementary Information S1.1 for a brief description of the WCS)^14,15^. Comparison of the cross-language dispersions (see Methods) between the real and randomised WCS data illustrated that the basic colour categories across languages are tightly clustered (having a significantly smaller dispersion value than the randomised languages)^3^. Similar distributions of the focal colours (see Methods) between the WCS languages and English implied the existence of universal forces shaping common colour categories across languages^4^. Though visualising the universality of linguistic colour categorisation pattern, these data-driven, statistical analyses ignore the roles of individual and socio-cultural factors in linguistic colour categorisation^9^.

Psychological experiments on human adults and/or children have focused on individual learning or perception of colour terms^6,16,17^. However, many experiments concentrated on discrimination of colour stimuli around category boundaries, and lacked a proper manipulation of individual learning or perceptual constraints. Therefore, these studies failed to explain why certain stimuli tended to become focal colours across languages or whether individual factors play any dominant role in shaping linguistic colour categorisation patterns within and between languages.

Computational simulation^18,19^ has recently joined the endeavour to tackle these limitations, by tracing the emergence and spread of linguistic categories among individuals during socio-cultural transmissions^20–24^. Some models used predefined focal colours^22,23^ or pseudo stimuli from the hue space^12,24–26^. For example, the category game model^24^ incorporated a nonlinear curve of Just Noticeable Difference (JND, the lowest perceptual distance between colour stimuli that normal human eyes could discriminate) in the hue space. The comparison of the cross-language dispersions between the categories emergent under the human JND and a uniform JND resembled the one between the real and randomised WCS data^12^. Follow-up studies using the same model attempted to ascribe the emerging sequence of focal colours to the non-uniformity of the human JND^25^, and the cross-language categorical variations to the cultural histories of communities^26^.

However, the uni-dimensional property of hue and the JND curve therein only reflect one aspect of perceptual characteristics of colours. In addition, it is non-trivial to extend from the hue space to the multi-dimensional colour perceptual space^27^, the perceptual constraints on colours are actually an integrative function of JNDs in multiple dimensions^28^, and it is very difficult to quantify such JNDs exhaustively in the whole colour space using empirical experiments.

Other models adopted real stimuli to reflect the perceptual constraints on humans. For example, some models directly recruited the 330 Munsell chips used in the WCS data collection^13,22^, but these highly saturated, environmentally infrequent stimuli^8^ are insufficient to capture the irregularity of colour perception in the whole colour space^11,29^. Only a few studies adopted a rich set of stimuli^20^ extracted from real pictures. These studies demonstrated that rather than the non-uniformity of JNDs the non-uniformity of perceptual distances between colour stimuli is sufficient to trigger different linguistic colour categories. However, due to a lack of proper control of repetition of colour stimuli, they failed to separate the roles of perceptual constraints (which are denoted by the displacement of stimuli and relative distances between them in the perceptual space) and stimulus frequency (which make distinctions of some stimuli more or less frequent or important than others) in shaping linguistic categorisation patterns.

**Figure 1:**
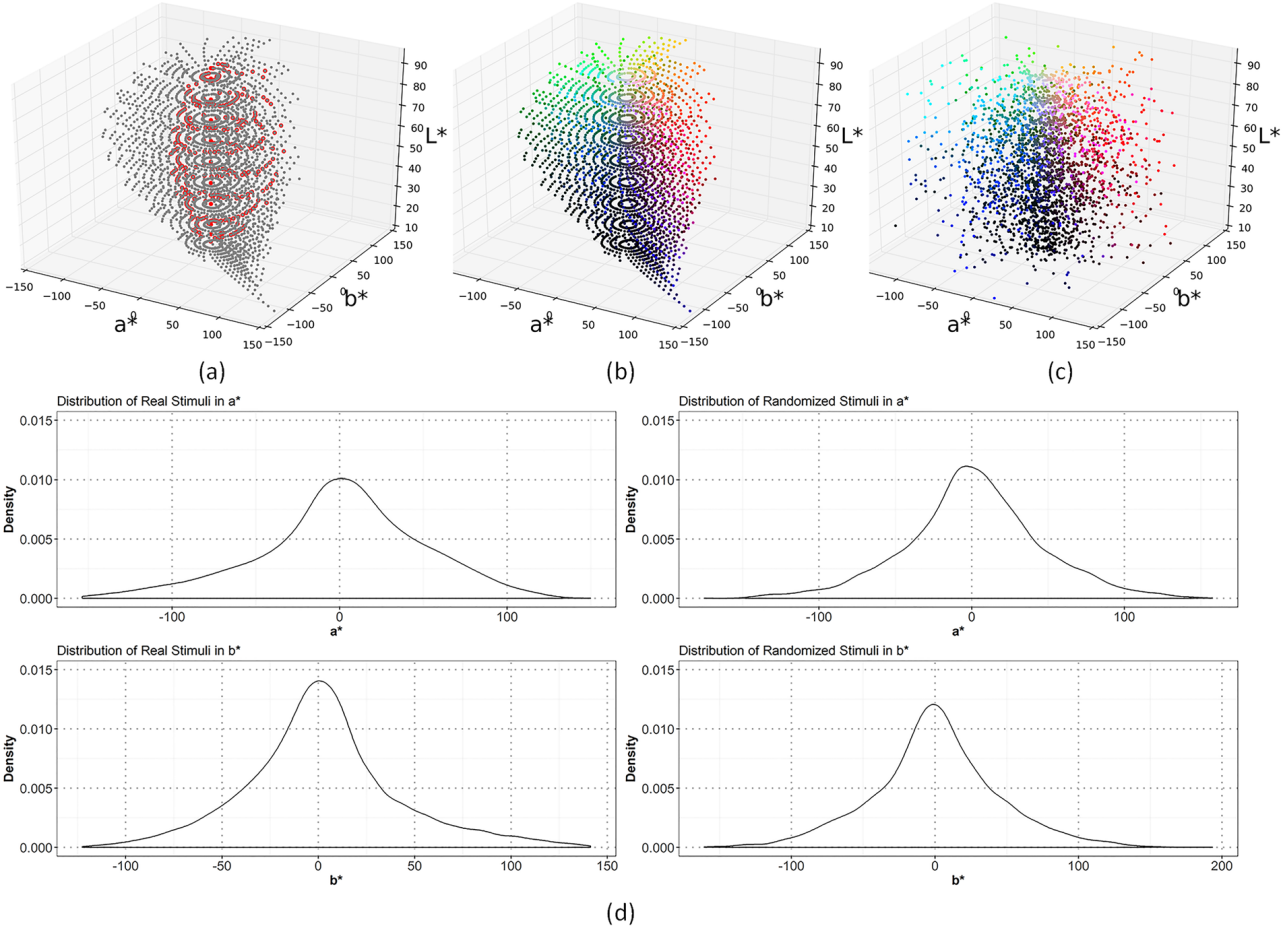
Displacements of the 2734 real colour stimuli (**a**,**b**) and one set of 2734 randomised stimuli (**c**) in the CIE L^*^a^*^b^*^ space. Red dots in (**a**) mark the 330 stimuli used in the WCS data collection, which constitute a subset of the 2734 real stimuli. Colours in (**b**,**c**) are based on the RGB values calculated from the CIE L^*^a^*^b^*^ coordinates of the real and randomised stimuli. Randomization in (**c**) is achieved by rotating each of the real stimuli along the L^*^ axis to a random degree. (**d**) shows that the real and randomised set of stimuli exhibit similar distributions respectively in the a^*^ (the two panels in the first row) and b^*^ axes (the two panels in the second row), but the displacement and relative distances of these stimuli are distinct as illustrated in the three-dimensional space (**a**,**c**).

Our study aimed to address the relative importance of perceptual constraints and socio-cultural transmissions to linguistic colour categorisation. We adopted a rich set of distinct colour stimuli perceivable to normal human eyes (see Methods). These physical stimuli were originally listed in a large-scale, psychophysical experiment^30^ aiming to amend the inconsistencies between colour stimuli and their corresponding chips in the visually-uniform Munsell Colour Order System. The total 2734 stimuli in this psychophysics dataset are distributed non-uniformly in the International Commission on Illumination (CIE) L^*^a^*^b^*^ space (see Fig. 1(a,b)). Unlike the uni-distance Munsell Colour System in hue, saturation and lightness, the CIE L^*^a^*^b^*^ system segments the perceptual space based on the degree of visual angle along the lightness dimension. In this way, the nonlinear relations of L^*^, a^*^, and b^*^ best mimic the nonlinear response of human eyes towards colours^27^. These stimuli constitute the physical foundation of linguistic colour categories, and the perceptual distances between them (quantified by the CIE94 equations^27^, see Supplementary Information S1.2 for the calculation of such distance) reliably indicate their perceptual dissimilarity to human eyes. Compared to the JND curve in the hue or other uni-dimensional perceptual space, the physical displacement of these stimuli in the three-dimensional CIE L^*^a^*^b^*^ space reflects more comprehensively the human perceptual constraints towards colours.

We designed an agent-based^18,19^, exemplar-based categorisation^31,32^ model; artificial agents tried discriminating colour stimuli by associating them into categories with distinct colour terms. During iterative, pair-wised linguistic communications, whenever agents failed to use common terms to call some stimuli, they automatically associated these stimuli respectively into new categories having distinct terms; whenever they reached mutual understanding by calling a stimulus a specific term, they discarded competing terms in their categories associating this stimulus to ensure future mutual understanding based on that term (see Supplementary Information S1.3 for model details). Without global supervision, this local learning strategy fulfilled the functional goal of socio-cultural transmissions, bridged the gap between perception and language, and required no manipulating parameters.

To differentiate the roles of perceptual constraints and socio-cultural transmissions in linguistic colour categorisation, we conducted two sets of simulations, under respectively the 2734 real stimuli from the psychophysics dataset and 2734 randomised stimuli (also see Supplementary Information S1.5). The numbers of stimuli in the two sets of simulations were the same in order to compare the dynamics of linguistic colour categorisation during socio-cultural transmissions. Inspired by^3^, the randomised stimuli in our study were created by rotating, along the L^*^ axis, each of the real stimuli to a random degree (see Fig. 1(c) and Methods). Not greatly changing the stimuli distributions in the a^*^ and b^*^ axes (see Fig. 1(d)), this rotation altered the displacement of stimuli and their relative distances in the whole perceptual space, thus distorting the human perceptual constraints on colours. Unlike Kay & Regier’s study^3^, we rotated the raw colour stimuli, rather than empirically observed focal colours in different languages. Agents gradually developed their colour categories and focal colours from the two sets of stimuli that reflect human perceptual constraints and not. There were 110 runs in each set, matching the number of languages in the WCS. We then compared the categorisation pattern shown in the WCS with the emergent ones obtained in the two sets of simulations, based on some well-attested measures such as the cross-language dispersion and focal colour distribution. Though borrowing these measures, our study was not limited to the focal colours in the WCS; instead, it further tested whether focal colours could gradually emerge from different sets of colour stimuli that reflected human perceptual constraints and not, and whether the emergent focal colours quantitatively resembled those in the WCS, as indicated by the adopted measures. We also conducted simulations under three distinct population sizes (50, 100, and 200 agents) to illustrate the generality of the results.

## Results

### Dynamics of linguistic colour categorisation

**Figure 2:**
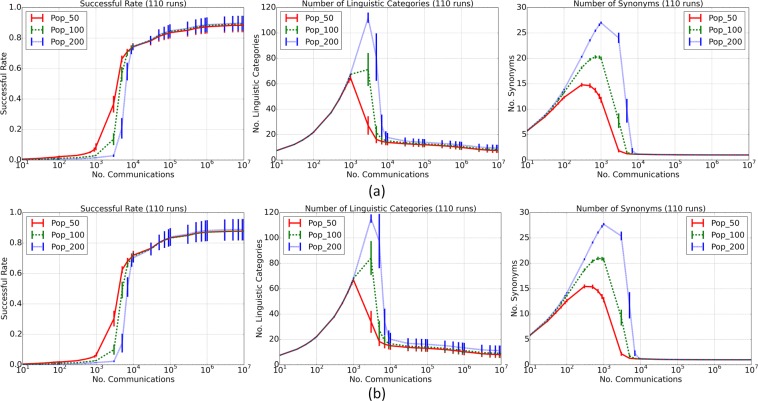
Dynamics of linguistic colour categorisation in the two sets of simulations under the real (**a**) and randomised (**b**) stimuli. Different curves correspond to the results under different numbers of agents (50, 100, and 200). Results in each set are averaged over 110 runs. Error bars denote two times of standard errors. Dynamics is illustrated by successful rate (the left panels in (**a**,**b**)), number of linguistic categories (the middle panels), and number of synonymous colour terms (the right panels).

Under the two distinct sets of colour stimuli, the two sets of simulations exhibited similar linguistic categorisation dynamics (see Fig. 2), as evident by the S-shape increase in successful rate (proportion of communications whereby the hearer’s guess matches the topic described by the speaker) and three-phase (increasing, dropping, and stabilizing) dynamics in the numbers of linguistic categories and synonymous terms (colour terms that encode more than one stimulus) (see Supplementary Information S1.4 for the detailed description of the dynamics). This dynamics resembled the one in the category game model^24^, and depended less on the numbers of agents and colour stimuli (see Extended Data Fig. 1 in Supplementary Information). These results indicated that even under different sets of colour stimuli socio-cultural transmissions played a common role of generating shared linguistic categories among individuals for efficient exchange of colour stimuli.

**Figure 3:**
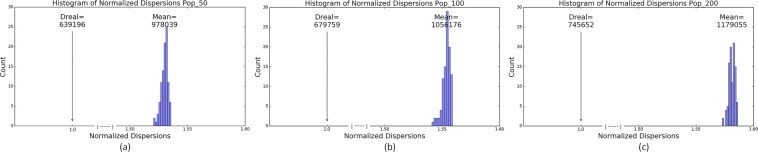
Comparisons between the cross-language dispersion of the emergent languages under the 2734 real stimuli (denoted by *D*_*Real*_) and the cross-language dispersions of 100 sets of languages each containing 110 random languages (${D}_{Rand}^{^{\prime} }$). Emergent languages were obtained at 10^7^ communications per agent (the linguistic system then stays in a relatively stable phase, see Supplementary Information S1.4 for the description of the linguistic categorisation dynamics of the model). (**a**–**c**) show comparison results under different population sizes (50 agents in (**a**), 100 in (**b**), and 200 in (**c**)). In a figure, the x-axis is normalised over ${D}_{Real}$, the arrow denotes the position of ${D}_{Real}$, the value of which is shown above the arrow. The value after “Mean” is the mean ${D}_{Rand}^{^{\prime} }$ over the 100 sets of random languages.

The cross-language dispersion of the emergent languages under the real stimuli (denoted by *D*_*Real*_) was significantly smaller (*p* < 0.01) than that under the randomised stimuli (*D*_*Rand*_) (*D*_*Real*_:*D*_*Rand*_ = 1:1.401 for 50 agents, 1:1.478 for 100 agents, and 1:1.559 for 200 agents). In addition, *D*_*Real*_ was significantly smaller (*p* < 0.01) than the cross-language dispersion (*D*′_*Rand*_) of 100 sets each containing 110 random languages created by rotating the focal colours of the emergent languages under the real stimuli (see Fig. 3). *D*_*Real*_ was also significantly smaller (*p* < 0.01) than the cross-language dispersion (${D}_{Rand}^{^{\prime\prime} }$) of another 100 sets each containing 110 languages obtained under a distinct set of randomised stimuli (see Extended Data Fig. 2).

These results demonstrated that the emergent categories under human perceptual constraints (reflected by the real colour stimuli) were clustered more closely than those under different sets of randomised colour stimuli. This extended Kay & Regier’s study^3^ only showing that focal colours from human languages were clustered more closely than randomized focal colours. Different dispersion ratios between our study and Kay & Regier’s study was due to different ways of randomising the real stimuli and recorded focal colours in the WCS. Similarly big ${D}_{Rand}$, ${D}_{Rand}^{\text{'}}$, and ${D^{\prime\prime} }_{Rand}$ under the same number of agents also indicated that randomising emergent focal colours under the real stimuli and directly randomising real stimuli to obtain emergent focal colours had similar roles in distorting the human perceptual constraints towards colours and the consequential emergent categorisation patterns. Note that the dispersion values slightly increased in bigger populations. This was due to the variations induced by many agents during socio-cultural transmissions. The degree of such variations increases along with the number of agents. Nonetheless, such variation could not influence the general finding that the dispersions of the emergent colour categories under the real stimuli are significantly lower than those under the randomised stimuli.

### Emergent focal colours

**Figure 4:**
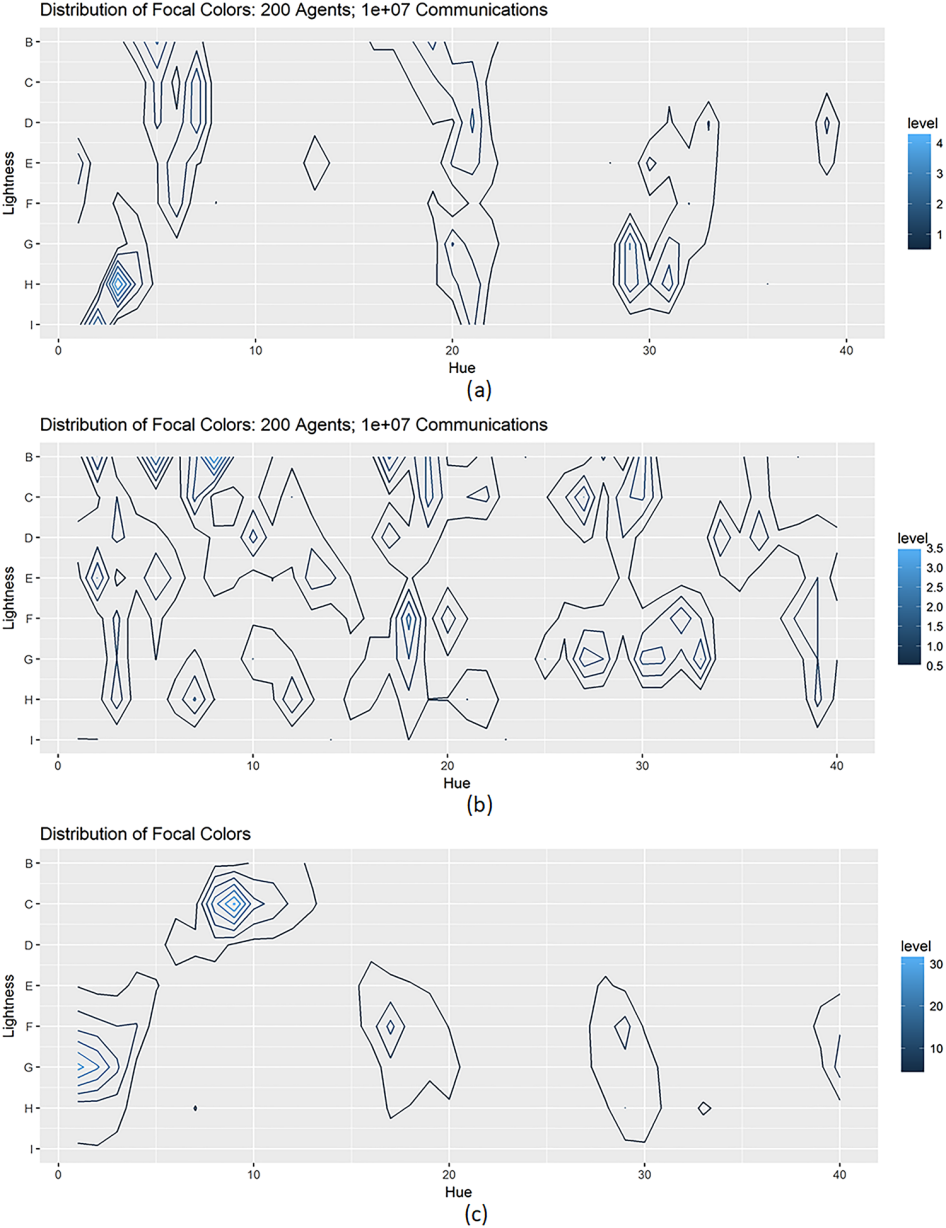
Focal colour distributions based on the 2734 real stimuli (**a**), the 2734 randomised stimuli (**b**), and the WCS data (**c**) (reproduced from the early study^4^). In each simulation, the population contained 200 agents. Distributions were drawn at 10^7^ communications per agent. Results were obtained from 110 runs in each set of simulations. Clusters in (**a**) are distinct from those in (**b**), but resemble the ones in (**c**) with slight coordinate mismatch. The coordinate mismatch and level differences between the clusters in (**a**,**c**) were due to different numbers of stimuli (2734 in (**a**) vs. 330 in (**c**)) used to draw the plots, distinct distance measures (CIE94 in (**a**) and Euclidean distance in (**c**)), and the normalisation operation in (**a**) during the projection of stimuli from the CIE L^*^a^*^b^*^ space to the Munsell colour stimuli array (see Methods).

Despite of the similar dynamics of linguistic categorisation, the emergent categorisation patterns in the two sets of simulations were starkly distinct, as revealed by the focal colour distributions in the Munsell colour stimulus array that contains the 320 Munsell colour chips used for the WCS data collection (together with the 10 chips at different grey levels) (see Fig. 4(a,b)).

Under the real stimuli, the emergent focal colours constructed several clusters (see Fig. 4(a) for the mean results in 200-agent populations). Many clusters lay around those formed by the WCS languages (see Fig. 4(c)), and resembled the focal colours *red*, *yellow*, *green*, and *blue* (as well as *black* and *white*)^5^ in English. These clusters were consistent with the focal colours that pre-linguistic infants could parse using their recognition memory^33^. The peak-to-peak distance (see Methods) between Fig. 4(a,c) was 14.628 (also see Extended Data Table 1 for the gradual drop of this measure from 10^6^ to 10^7^ communications per agent, which indicates that under the real colour stimuli, the emergent focal colours become more and more similar to the focal colours in the WCS languages). Similar results were obtained in the simulations under other conditions (see Extended Data Figs 3 and 4 for the emergent colour categories under different numbers of agents and communications).

By contrast, under the randomised stimuli, the emergent focal colours obtained at the same number of socio-cultural transmissions were distributed sparsely, and did not resemble those in the WCS languages (see Fig. 4(b) for the mean results in 200-agent populations). The peak-to-peak distance between Fig. 4(b,c) (21.475) was larger than that between Fig. 4(a,c) (14.628) (also see Extended Data Table 2 for the fluctuation of the peak-to-peak distance from 10^6^ to 10^7^ communications per agent under the randomised colour stimuli. This indicates that the emergent languages would never generate a categorisation pattern resembling that in the WCS). In addition, these emergent focal colours under the randomised stimuli differed greatly from those under the real stimuli (see Extended Data Table 3 for the comparison of the peak-to-peak distance between the emergent focal colours under the real stimuli and those under the randomised stimuli). Furthermore, the emergent focal colours under different sets of randomised stimuli were distinct not only from each other but also from the WCS data (see Extended Data Fig. 5 for the emergent colour categories under different sets of randomised colour stimuli).

The two sets of simulations differed only in the displacements of the 2734 colour stimuli in the perceptual space, which reflected distinct perceptual constraints towards colours (perceptual distances between pairs of stimuli are different). Therefore, the comparison of focal colour distributions between the two sets of simulations illustrated that: it was perceptual constraints towards colours that determined the emergent categorisation patterns; and if such constraints reflect the human perceptual constraints on colours, the emergent categorisation pattern would largely resemble the one as evident in the WCS.

## Discussion

Perception is inherent in linguistic colour categorisation; what human eyes can perceive and discriminate affect emergent linguistic descriptions of colour stimuli. Socio-cultural transmissions are also essential, which provide the means of transcription of perceptual constraints into linguistic colour categories. During transmissions, apparent cross-language or cross-culture diversity occurred inevitably, due to individual differences (or socio-cultural factors, see Supplementary Information S1.5).

Our study directly compared the empirical data in the WCS with the simulation results obtained under the human and randomised perceptual constraints on colours. It explicitly illustrated that: differentially distributed stimuli in the three dimensional colour perceptual space reflected distinct perceptual constraints towards colours; and as a result, the emergent linguistic categorisation patterns became distinct, though following a similar process of linguistic categorisation under the same socio-cultural setting.

Our study also showed that socio-cultural transmissions could not override the role of perceptual constraints in shaping linguistic categorisation patterns during discriminative communications as simulated in our model. These results demonstrate how human perception and socio-cultural factors interact with each other to trigger linguistic universality. They also serve as decisive evidence that: the universality in linguistic colour categorisation is not constructed arbitrarily by language or socio-cultural factors; instead, it is organised and constrained primarily by perceptual constraints on colours.

This study adopted a set of physical colour stimuli covering the whole multi-dimensional perceptual space, and allowed direct manipulation of artificial agents’ perceptual constraints towards colours. It extends the existing human experiments and simulations that failed to adjust human perceptual constraints or were based primarily on non-exhaustive, biased stimuli.

The dominance of perceptual constraints over socio-cultural factors in the domain of linguistic colour categorisation can also shed important lights on the general discussion of relations between human perception, language, and socio-cultural environment. For example, it challenges the Sapir-Whorfian hypothesis that language affects human perception^34^. Our study indicates that at least in the domain of linguistic colour categorisation during simple communications, human perceptual constraints on colours still cast the determinant role in prescribing the focal regions around which linguistic colour categories are formed, whereas language or other socio-cultural factors only lead to variations in boundaries of colour categories across languages. These variations could affect the individual perceptual discrimination decisions on colour stimuli lying around mixture boundaries of languages, as illustrated in previous psychological or neurological experiments^35–39^, but the focal regions of the basic colour categories of those languages remain largely overlapped.

In addition, our conclusion is based on unsupervised, local learning during simple pair-wised communications. A recent study pointed out that strong culture-specific or usage-based influence might induce variation on the number of colour terms across languages, or cause communities to favour linguistic categories of some colours (e.g., red) over others^40^. Nonetheless, perceptual influence such as discriminating warm and cool colours still takes effect in naming objects and backgrounds.

Furthermore, the dominance of perceptual constraints on linguistic categories also inspires investigations on causal factors of other categorical linguistic components, such as the linguistic categories of other visual properties like shapes^41^ or phonological properties like vowels^42^ or lexical tones^43^. Many components as such also resulted from close interactions between perceptual and socio-cultural factors. This line of research could shed light on the discussions about the foundations of human language^44^. Apart from human experimental studies, agent-based models as in our study could help to clarify the relative importance of various factors to the evolutionary process of linguistic categorisation and emergent universality therein.

Our study integrates the empirical data from psychophysics and linguistics. Incorporating psychophysics datasets and simulation approach into the linguistic or psychological research on colour categorisation extended existing studies focusing exclusively on the limited WCS dataset. A better understanding of the universality (and diversity) of linguistic colour categorisation requires attentions to both linguistic typology of colour categories and psychophysics research of human colour perception. As for typology, due to irregular mappings between the stimuli in the three-dimensional CIE L^*^a^*^b^*^ perceptual space and those in the Munsell colour stimulus array^7,11^, a set of colour stimuli richer than the 330 Munsell chips could lead to a better documentation of colour categories. This documentation could reflect not only the detailed boundary variations across languages but also the effects of human perceptual constraints on shaping linguistic colour categories around certain perceptual regions. As for psychophysics studies of colour perception, to better reflect human perceptual constraints, it is necessary to take into account the influence of individual linguistic backgrounds on discrimination of colour stimuli. Only in this interdisciplinary manner can we better understand why certain colours tend to become focal colours in human languages, how similar distributions of focal colours gradually emerge, and what is the relation between perceptual features of colour stimuli and linguistic colour categorisation^44,45^.

## Methods

### Colour stimuli perceivable to human eyes

The physical colour stimuli used in our study were extracted from the Colour Science Laboratory (https://www.rit.edu/cos/colorscience/rc_munsell_renotation.php). These 2734 stimuli were represented in the CIE *xyY* space. Their chromaticity coordinates were calculated using illuminant C and the CIE 1931 2-degree observer. We used the CIE L^*^a^*^b^*^ coordinates of the colour stimuli in the simulations (see Supplementary Information S1.2 for the coordinate transition equations). Data collection in the WCS was conducted using the 330 Munsell chips (see Fig. S1(a)), which formed a subset of the total stimuli. Although the 330 chips could reliably record the major colour categories of a language, they were distributed sparsely in the whole colour perceptual space (CIE L^*^a^*^b^*^ space), by contrast, the total 2734 stimuli were widely spread in the perceptual space (see Fig. 1(a)).

In terms of quantity, this set of stimuli were much smaller than the one used in a modelling study^20^, which incorporated 25000 repeatable stimuli extracted from real photos. Due to repetition, this richer set of stimuli involved incidentally frequency information of stimuli, thus making it hard to disentangle the role of perceptual constraints on colours and that of stimulus frequencies in shaping linguistic colour categories. By contrast, our study adopted the 2734 distinct stimuli to address whether perceptual constraints on colours as reflected by the displacement of those stimuli in the perceptual space could predominately shape the universal colour categorisation pattern across languages. After this fundamental issue is resolved, we can carry out follow-up investigations addressing in particular the effect of stimulus frequency on linguistic colour categorisation.

### Cross-language dispersion

This measure was proposed by Kay & Regier in a statistical analysis of the WCS data^3^. They transformed the most representative Munsell chip(s) for each colour category (i.e., focal colour(s)) in each language in the WCS into the point(s) in the CIE L^*^a^*^b^*^ space. They then used Euclidean distance (see Eq. 1) to approximate perceptual distance between these points, and calculated cross-language dispersion *D* of a set (*S*) of languages, using Eq. 2:

1 $${\rm{\Delta }}E=\sqrt{{({L}_{1}^{\ast }-{L}_{2}^{\ast })}^{2}+{({a}_{1}^{\ast }-{a}_{2}^{\ast })}^{2}+{({b}_{1}^{\ast }-{b}_{2}^{\ast })}^{2}}$$

2 $$D=\sum _{{l}_{1},{l}_{2}\in S}\sum _{{c}_{1}\in {l}_{1},{c}_{2}\in {l}_{2}}\,\min \,{\rm{\Delta }}E$$

where *∆E* calculated the Euclidean distance between the focal colours of two categories *c*_1_ and *c*_2_ (their coordinates in the CIE L^*^a^*^b^*^ space are (${L}_{1}^{\ast },{a}_{1}^{\ast },{b}_{1}^{\ast }$) and (${L}_{2}^{\ast },{a}_{2}^{\ast },{b}_{2}^{\ast }$)) respectively from two languages *l*_1_ and *l*_2_ in *S*. *D* calculated the accumulated minimum distance between every category from any language and every category from any other language in *S*. The accumulative nature of *D* makes it subjective to heterogeneity at the individual and/or community level.

To make *D* meaningful, they created 1000 additional sets, each containing 110 languages created by rotating, along the L^*^ axis, the focal colour points in the original set *S* to a fixed random angle. This way of random rotation partially destroyed the categorical displacement in different languages. They then calculated the values of *D* in these sets. A comparison between the *D* of the WCS data and those of the randomised sets revealed that the *D* of the former was significantly smaller than the mean of the latter, indicating that the linguistic colour categories in the WCS were closely clustered^3^.

Our study borrowed the measure of cross-language dispersion, and treated the emergent colour categories in each run of the model as the colour categories of a “language”. Measuring focal colours in the simulation data proceeded as follows. For the data of a specific run (language), we first calculated the average number of distinct colour terms (*M*) used by the whole population. This value corresponded roughly to the number of emergent focal colour clusters in that run. Here, a focal colour was defined as the centroid of all stimuli using the same term. If the centroid was not a stimulus in the input set, the real stimulus in the input set having the smallest perceptual distance to the centroid was used as the focal colour. This setting was in line with the perceptual constraints on colours (i.e., eyes could only discriminate stimuli that their perceptual constraints allowed them to perceive). For each of the 2734 stimuli, we measured the proportions of agents who assigned the stimulus as a focal colour, normalized the proportion values to ensure the sum to be 1.0, and sorted the stimuli based on their normalized proportion values of being focal colours. Then, we chose the first *M* stimuli with high proportions as the focal colours.

After obtaining the emergent focal colours in the simulations, we conducted three comparisons based on the measure of cross-language dispersion. First, we compared the *D* of 110 emergent “languages” (runs) obtained under the 2734 real stimuli with the *D* of 100 (corresponding to a *p* value 0.01 during comparison) language sets, each containing 110 random “languages” created by randomising the focal colours of the 110 emergent “languages” under the real stimuli. Randomisation followed exactly Kay & Regier’s work^3^. The *D* of the emergent languages under the 2734 real stimuli were expected to be significantly smaller than the *D* of the randomised languages, just like what was shown based on the real and randomised WCS data.

Second, we compared the *D* of 110 emergent “languages” obtained under the 2734 real stimuli with the *D* of 100 additional sets, each containing 110 emergent “languages” obtained under a different set of 2734 randomised stimuli. Each set of stimuli was created using the same rotation method as to the creation of the 2734 randomised stimulus in Fig. 1(b). Rotation of each stimulus follows a different random angle, the degree of randomisation as such is higher than that of rotation of focal colours as in^3^, in which all focal colours from a run (language) are rotated to the same random angle. The *D* of the emergent languages under the 2734 real stimuli was expected to be significantly smaller than the *D* of the emergent languages obtained under different sets of 2734 randomised stimuli.

Third, we compared the *D* of the 200 language sets created by the above two ways of randomisation: The first 100 sets were created by randomising the focal colours of the emergent languages obtained under the 2734 real stimuli, and the second 100 sets were created by randomising the real stimuli and then obtaining the emergent languages under the randomised stimuli. This comparison aimed to check whether the two ways of randomisation were essentially the same in terms of distorting the human perceptual constraints on colours and the emergent categorisation patterns.

The WCS data were obtained based on the 330 Munsell chips and the Euclidean distance measure, whereas the simulation data in our study were obtained based on the 2734 stimuli in the CIE L^*^a^*^b^*^ space and the CIE94 equations (*∆E*_94_, see Supplementary Information S1.2). Together with individual differences as reflected by different numbers of agents in the population, all these factors induced the observed value differences between our study and the early work, but the expected outcome of comparison would be similar.

### Focal colour distribution

To visualise whether colour categories were organised around universally best examples across languages, Regier *et al*.^4^ extracted all focal colours from all informants of the 110 languages in the WCS, and calculated how many focal colours fell on each chip in the colour stimulus array (see Fig. 3(c)). By aggregation, they drew a contour plot on the colour stimulus array to record the numbers that each of the Munsell chips was chosen as a focal colour in the WCS languages^3,4^. This plot revealed several clusters around the English focal colours *red*, *green*, *yellow*, *blue*, as well as *black* and *white* (the last two colours could not be shown in the plot based on the 320 Munsell colour chips), which demonstrated that the focal colour commonalities were not limited to languages of industrialized societies and the chips within those clusters could be considered as universal foci.

Our study borrowed the same routine to draw focal colour distribution in the Munsell colour stimulus array using the focal colours of emergent categories in simulations, and compared the aggregated clusters of focal colours between the 110 languages in the WCS and the 110 emergent “languages” (runs) under the real or randomised colour stimuli. Following Kay & Regier’s method^3^, locating the Munsell chip mapping a point *p* in the CIE L^*^a^*^b^*^ space was done in two steps. First, we located the row *r* in the colour stimulus array whose L^*^ value was the closest to the L^*^ value of *p* (the L^*^ value of each row in the colour stimulus array is constant). Second, we chose the achromatic chip (white to black) and the chromatic chip in the row *r* that have the angle ${C}^{\ast }={\rm{atan}}(\frac{{b}^{\ast }}{{a}^{\ast }})$ closest to that of *p*. From the two chips, we selected the one having the radius ${H}^{\ast }=\sqrt{{a}^{\ast 2}+{b}^{\ast 2}}$ closest to that of *p*.

The mapping between the stimuli in the CIE L^*^a^*^b^*^ space and those in the Munsell colour stimulus array is apparently irregular^11^; many stimuli in the CIE L^*^a^*^b^*^ space end up being projected to the same Munsell chips. This is another reason that the stimuli in the two-dimensional Munsell colour stimulus array (and the uni-distance Munsell color space) cannot comprehensively reflect the human perceptual constraints on colours, compared to the CIE L^*^a^*^b^*^ space. Noting this, when projecting emergent focal colours from the CIE L^*^a^*^b^*^ space to the Munsell colour stimulus array, we first calculated how many real or randomised stimuli were mapped to each of the 330 Munsell chips (different sets of randomised stimuli would lead to different outcomes). Then, we used these values to normalize the frequency for each Munsell chip to be a focal colour. This normalisation induced level differences and slight coordinate mismatch for some stimuli during the comparison of the simulation results with the WCS data (the focal colour distribution based on the WCS data was replicated from the early works^3,4^), but it was necessary for more accurate analyses.

### Peak-to-peak distance

Inspired by the measure of dispersion^3^, we proposed the measure of peak-to-peak distance to compare the similarity between the emergent focal colours under the real or randomised stimuli and the focal colours in the WCS languages. Here, peaks were extracted from the contour plot at the Munsell colour stimulus array that contained 320 Munsell colour chips. A peak was a colour chip, the value on which was bigger than those of the four block neighbouring chips (note that the chips at the borders of the colour stimulus array were not considered as peaks). For the contour plot of the WCS languages, the value on a colour chip was the number of languages using a colour stimulus projected to this chip as a focal colour. For the contour plots of the emergent languages under the real or randomised colour stimuli, the value on a colour chip was the number of languages using a colour stimulus projected on this chip as a focal colour divided by the total number of colour stimuli that could be projected on this chip (see the above section). All identified peaks in this way were sorted based on their values (from big to small), and the first *N* peaks were chosen to calculate the peak-to-peak distance. For the WCS languages, *N* = 4, corresponding to the four focal colours *red*, *green*, yellow, and *blue*, respectively. For the emergent languages in the simulations, at a particular number of communications per agent, *N* was set to the average integer number of linguistic categories in the population minus 2 (excluding the categories for *black* and *white*).

The peak-to-peak distance between two sets of peaks identified in this way was based on the Euclidean distance at the two-dimensional colour stimulus array. It was calculated as in Eq. 3, in which W_P_, W_Q_ and ∆E_p,q_ were calculated as in Eq. 4:

3 $$peak \mbox{-} to \mbox{-} peak\,distance={W}_{P}\sum _{p\in P}mi{n}_{q\in Q}{\rm{\Delta }}{E}_{p,q}+{W}_{Q}\sum _{q\in Q}mi{n}_{p\in P}{\rm{\Delta }}{E}_{p,q}$$

4 $${W}_{p}=\frac{size(P)}{size(P)+size(Q)},\,{W}_{Q}=\frac{size(Q)}{size(P)+size(Q)},\,{\rm{\Delta }}{E}_{p,q}=\sqrt{{({h}_{p}-{h}_{q})}^{2}+{({l}_{p}-{l}_{q})}^{2}}$$

where *P* and *Q* were the two sets of peaks for comparison. *size*(*P/Q*) is the number of peaks in that set. The weights were added to balance the influence of different numbers of peaks in the two sets. *p* and *q* were peaks respectively from the two sets, and *h*_*p*_ and *l*_*p*_ were the coordinates in hue and lightness of a peak *p*. The smaller the peak-to-peak distance, the more similar the distributions of the two sets of focal colours. A gradual drop of the peak-to-peak distance along with the increase in the number of communications per agent also indicates whether along with the cultural evolution the distributions of the two sets of focal colours would become more and more similar to each other.

## Supplementary information

Supplementary Information
