# Supplementary material for: Perceptual constraints on colours induce the universality of linguistic colour categorisation
Source: Sci Rep. 2019 May 22;9:7719. doi: 10.1038/s41598-019-44202-6 (PMC6531495; doi:10.1038/s41598-019-44202-6)
Supplement: Supplementary file 1 — Supplementary Information [file 41598_2019_44202_MOESM1_ESM.docx]

##### Perceptual constraints on colours induce the universality of linguistic colour categorisation

Tao Gong, Hangxian Gao, Zhen Wang, Lan Shuai

##### Supplementary Information

**Sections**

**S1. Supplementary Text**

**S1.1. World Colour Survey (WCS)**

**S1.2. Coordinate transformation and perceptual distance calculation of colour stimuli**

**S1.3. Colour categorisation model**

**S1.4. Dynamics of the model**

**S1.5. Reasons for not manipulating socio-cultural transmissions**

**S2. Extended Data Figures and Tables**

**S3. Additional References**

**S1. Supplementary Text**

**S1.1. World Colour Survey (WCS)**

Kay and Berlin conducted the first survey on 20 languages in 1969 (*14*), and more data from more languages were collected from 1976 to 1980. The whole database was published online in 2003 at: <http://www.icsi.berkeley.edu/wcs>. It contained the basic colour categories and colour terms of 110 languages having no written forms and spoken in small-scale, non-industrialized societies. Data collection proceeded as follows. For each language, there were on average 24 native speakers as informants, who were asked to name each of the 330 colour chips in the Munsell colour stimulus array (see Figure S1(a)) and to report the best representative chip(s) for each colour term (see Figure S1(b) for the colour categorisation pattern of a language in the WCS). Among the chips, 320 encoded 40 hue gradations and maximum saturation, and 10 encoded *black*, *grey*, and *white* at 10 lightness levels. All the 330 chips were presented in a predefined random order to informants.


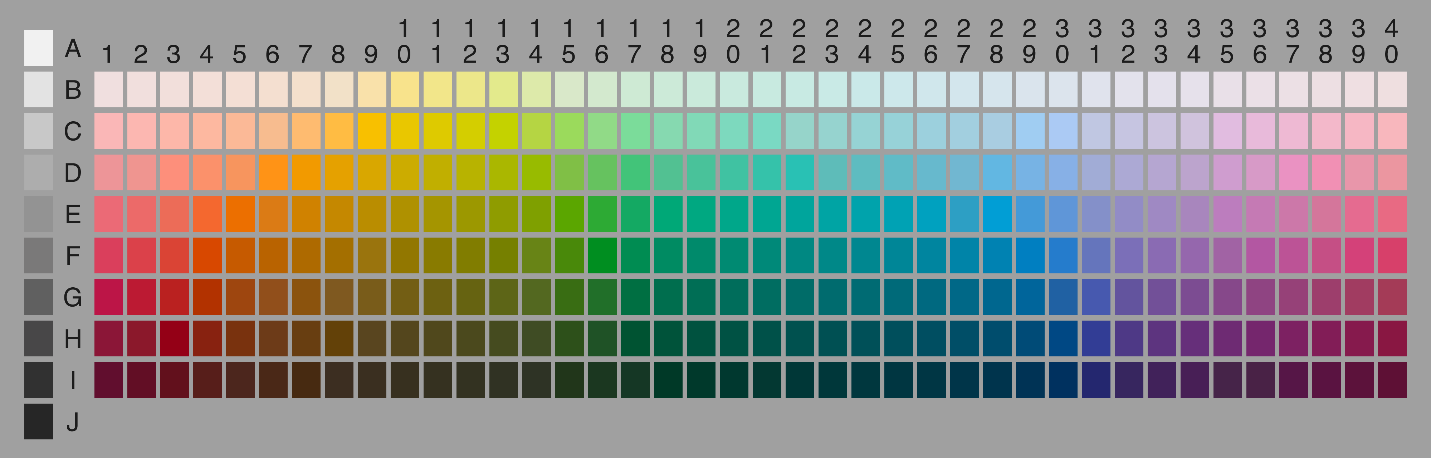


(a)


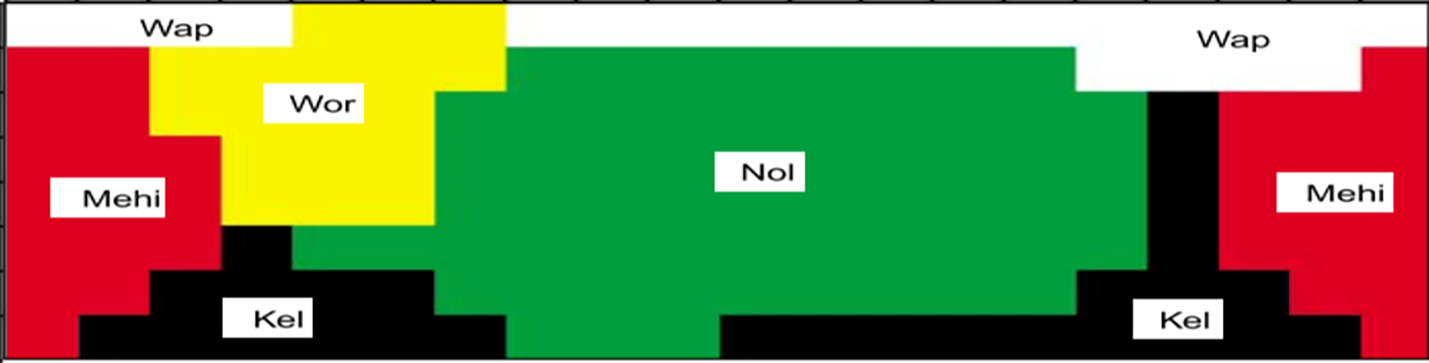


(b)

**Figure S1** (a): 330 colour chips in the Munsell colour stimulus array, including 320 colourful chips and 10 black/white/grey chips; (b): Colour categorisation pattern of a language, Bernimo, in the WCS (*46*). Colourful patches denote colour categories, whose terms are shown in text.

**S1.2. Coordinate transformation and perceptual distance calculation of colour stimuli**

We used the CIE *L^*^a^*^b^*^* coordinates of colour stimuli in the simulations. The CIE *L^*^a^*^b^*^* space is *perceptually uniform*. This does not mean that the perceptual distances between any two closely displaced colour stimuli are the same; instead, in this three-dimensional perceptual space, the same change in a colour value corresponds roughly to the same change in visual importance. Accordingly, the perceptual difference between any stimuli can be reasonably approximated by treating each stimulus as a point in this three-dimensional colour space and then calculating their perceptual distance.

Following the CIE standard (*27,47*), we first transformed the CIE *xyY* coordinates into the CIE *XYZ* coordinates, using Equation S1:

$\begin{matrix} X=\frac{xY}{y} \\ Y=Y \\ Z=\frac{\left( 1-x-y \right)Y}{y} \end{matrix}$ (S1)

Where *X*, *Y*, and *Z* on the left side were the CIE *XYZ* coordinates, and *x*, *y*, and *Y* on the right side were the CIE *xyY* coordinates. We then transformed the CIE *XYZ* coordinates into the CIE *L^*^a^*^b^*^* coordinates, using Equation S2:

$\begin{matrix} L=116f_{y}-16 \\ a=500(f_{x}-f_{y}) \\ b=200(f_{y}-f_{z}) \end{matrix}$ (S2)

Where *f_x_*, *f_y_*, and *f_z_* were calculated using Equation S3:

$f_{x}=\left\{ \begin{matrix} \sqrt[3]{x_{r}}\text{ }, \text{when }x_{r}>\varepsilon\\ \frac{Kx_{r}+16}{116}\text{, else} \end{matrix} \right., f_{y}=\left\{ \begin{matrix} \sqrt[3]{y_{r}}\text{ }, \text{when }y_{r}>\varepsilon\\ \frac{Ky_{r}+16}{116}\text{, else} \end{matrix} \right., f_{z}=\left\{ \begin{matrix} \sqrt[3]{z_{r}}\text{ }, \text{when }z_{r}>\varepsilon\\ \frac{Kz_{r}+16}{116}\text{, else} \end{matrix} \right.$ (S3)

Where $x_{r}=\frac{X}{X_{r}}, y_{r}=\frac{Y}{Y_{r}}, z_{r}=\frac{Z}{Z_{r}}$. *X*, *Y*, and *Z* were the CIE *XYZ* coordinates, *X_r_*, *Y_r_*, and *Z_r_* were the tri-stimulus values of the reference colour *white*, *ɛ* = 0.008856 and *K* = 903.3.

Perceptual distances between colour stimuli in the CIE *L^*^a^*^b^*^* space can be calculated in many ways, such as the Euclidean distance, the colour-difference formula BFD, and the equations provided by the CIE or Colour Measurement Committee (CMC). Among these measures, the CIE94 equations (*27,47*) appear to be more perceptually uniform than the others (*48*). We thus adopted the CIE94 equations to calculate perceptual distance (*∆E*_94_) between stimuli, as shown in Equation S4:

$\Delta E_{94}=\sqrt{{(\frac{{\Delta L}^{*}}{k_{L}S_{L}})}^{2}+{(\frac{\Delta C_{ab}^{*}}{k_{C}S_{C}})}^{2}+{(\frac{{\Delta H}_{ab}^{*}}{k_{H}S_{H}})}^{2}}$ (S4)

Where ($L_{1}^{*},a_{1}^{*},b_{1}^{*}$) and ($L_{2}^{*},a_{2}^{*},b_{2}^{*}$) are coordinates of the two stimuli *c*_1_ and *c*_2_ in the CIE *L^*^a^*^b^*^* space, and the other parameters are calculated as in Equation S5:

$\begin{matrix} \begin{matrix} {\Delta L}^{*}=L_{1}^{*}-L_{2}^{*} \\ {\Delta C}_{ab}^{*}=\sqrt{{a_{1}^{*}}^{2}+{b_{1}^{*}}^{2}}-\sqrt{{a_{2}^{*}}^{2}+{b_{2}^{*}}^{2}} \\ {\Delta H}_{ab}^{*}=\sqrt{{(a_{1}^{*}-a_{2}^{*})}^{2}+{(b_{1}^{*}-b_{2}^{*})}^{2}-{{\Delta C}_{ab}^{*}}^{2}} \end{matrix} \\ \begin{matrix} C^{*}=\sqrt{({a_{1}^{*}}^{2}+{b_{1}^{*}}^{2})({a_{2}^{*}}^{2}+{b_{2}^{*}}^{2})} \\ S_{L}=1; S_{C}=1+0.045\sqrt{C^{*}}; S_{H}=1+0.015\sqrt{C^{*}} \end{matrix} \\ k_{L}=k_{C}=k_{H}=1 \text{for reference coditions} \end{matrix}$ (S5)

Where *C^*^* was the geometric mean ensuring identical distances between *c*_1_ and *c*_2_ and between *c*_2_ and *c*_1_­. By definition, if *∆E*_94_ between two colour stimuli was smaller than 1.0, normal human eyes could not distinguish them (*48*). Note that the *∆E*_94_ between each pair of stimuli used in our study exceeded this minimum threshold.

In addition to our study, a recent simulation exploration also adopted a number of colour stimuli from the CIE *L^*^a^*^b^*^* space (*49*). However, that model uniformly segmented the colour space (100×200×200 in the CIE *L^*^a^*^b^*^* space) into a number of equally-sized (25×50×50) cubes, in each of which 4 stimuli were used for agents to develop colour categories. As shown in Figure 1 in the main text and the above discussion, the three-dimensional colour space is not cubic; instead, those perceivable colour stimuli to human eyes form an irregular space. In addition, although adopting standard equations (e.g., CIE2000) to calculate perceptual distances between stimuli, that model arbitrarily defined some parameters to reflect human eyes’ perceptual constraints on colours. By contrast, in our study, the non-uniform distribution of the total 2734 colour stimuli in the perceptual space directly reflected such constraints without requiring arbitrary parameters. Furthermore, due to involving arbitrary parameters and not using existing statistical measures as adopted and discussed in our study and previous ones (*3*,*4*), that model only generated some colour categories roughly matching the empirical data, e.g., the categories of cold and warm colours in human languages. Therefore, that model failed to make a more informative comparison between the simulation data and the WCS empirical data as in our study. Finally, that model focused on subjective learning of colour categories, rather than objective, unsupervised learning during discriminative communications as in our model. This made that model unable to illustrate and discuss how human perceptual constraints and socio-cultural transmissions interact with each other to trigger the universality in linguistic colour categorisation.

**S1.3. Colour categorisation model**

The model was inspired by the category game on the hue dimension (*24*), with significant extensions in many aspects. In our model, *N* individuals (artificial agents) communicated iteratively with each other in a pairwise fashion. In each communication, two agents (a speaker and a hearer) were randomly chosen from the population to talk about a scene. The scene contained *M* (≥2) stimuli randomly chosen from the full set of colour stimuli used in the simulation. *∆E*_94_ between any two stimuli in the scene must be greater than 1.0, such that agents could distinguish them (note that this condition always holds using our stimuli). One of the stimuli was the *topic* of the communication, and the other(s) was the *context*. To distinguish the topic from the context, agents needed to partition the stimuli represented in the CIE *L^*^a^*^b^*^* space within the Macadam limit into non-overlapping, contiguous segments called *perceptual categories*. Each perceptual category contained one stimulus (as the exemplar of the category) and an inventory of colour terms (linguistic descriptions of the stimulus). If two or more perceptual categories began to share a common term, they constituted a *linguistic category*, and the common term could be used to describe all the stimuli from those perceptual categories. Agents operated on perceptual categories, and linguistic categories emerged from scratch as abstract, high-level grouping of perceptual categories. Agents had no categories in their repertoires at the beginning of a simulation.

A communication proceeded in three steps: production, perception, and feedback.

*Production*: the speaker tried to discriminate the scene by assigning each stimulus in the scene to one of its perceptual categories whose exemplar had the smallest *∆E*_94_ to the stimulus. Here, there were three cases:

1. The topic was not associated into any category in the speaker’s repertoire. This often occurred at the early stage of a simulation. In this case, the speaker created a new category using the topic as its exemplar, put a new term to the inventory of the category, and transmitted the term to the hearer.
2. The topic was associated into a category, and the context stimuli in the scene were not associated into the same category. In this case, the speaker chose randomly a term in the inventory of the category that associated the topic, and transmitted the term to the hearer.
3. The topic and at least one context stimulus in the scene were associated into the same category. In this case, the speaker replaced the exemplar of the category with the topic, added a new term to the inventory of that category, and transmitted the term to the hearer. Meanwhile, as for each context stimulus associated into the same category with the topic, the speaker created a new category, which used the stimulus as the exemplar and inherited all the terms in the original category, plus a new term.

*Perception*: after receiving the term sent by the speaker, the hearer tried to identify a set of perceptual categories that could associate at least one stimulus in the scene and also contain the transmitted term. This perception process was also based upon *∆E*_94_ between each stimulus in the scene and the exemplar of each category in the hearer’s repertoire. Here, there were three cases:

1. The set was empty. This often occurred at the early stage of a simulation. In this case, the hearer pointed at nothing.
2. The set contained only one category whose exemplar had the smallest *∆E*_94_ to a single stimulus in the scene. In this case, the hearer pointed at the stimulus, referring to that stimulus as the topic. Note that such smallest *∆E*_94_ might or might not be smaller than 1.0. The hearer tried the best to perceive the heard term into a colour stimulus in the scene. If the smallest *∆E*_94_ was smaller than 1.0, it meant that the hearer’s eyes simply treated the stimulus in the scene to be identical to the exemplar of the category in the hearer’s repertoire. If the smallest *∆E*_94_ was greater than 1.0, the hearer could distinguish the two stimuli (the one in the scene and the exemplar of the category), but still treated the stimulus in the scene as the perceptually closest stimulus that the term could describe.
3. The set contained more than one category and the exemplars of these categories had the smallest *∆E*_94_ to different stimuli in the scene. In this case, the hearer tried guessing by pointing randomly at one of the stimuli associated into these categories, and treated that stimulus as the topic.

*Feedback*: after perception, the speaker unveiled the topic via pointing. If the stimulus pointed by the hearer matched the topic, the communication was deemed successful. Then, the speaker removed all the other terms but the transmitted one in the inventory of the category that associated the topic. As for the hearer, if the topic was assigned to a category and the context stimuli were not assigned to the same category, the hearer discarded all the other terms and kept the transmitted one in the inventory of the category. Otherwise, the hearer used the topic as the exemplar of that category, and the transmitted term as the only term in the inventory of that category.

If the stimulus pointed by the hearer did not match the topic, the communication was deemed failed. In this situation, if the topic could not be assigned to any of its categories, the hearer created a new category associating the topic, and added the transmitted term to its inventory. If the transmitted term was not in the category’s inventory, or the topic and one of the context stimuli were assigned to the same category, the hearer used the topic as the exemplar of that category, and added the transmitted term into its inventory. Per each context stimulus assigned to the same category, the hearer created a new category to associate it. A newly-created category would inherit all terms of the original category, plus a new one.

**S1.4. Dynamics of the model**

The categorisation mechanisms defined in our model were based upon comparing stimuli to be categorised with the exemplars of available categories. In line with the exemplar theory (*50-54*), these mechanisms have been argued to be more realistic than the prototype approach (*55-57*) in categorising sensory cues (*58-60*). In addition, these exemplar-based categorisation mechanisms required no controlling parameters. The whole model asked for only three parameters: *N* (number of agents), *C* (number of communications per agent), and *M* (number of stimuli in each scene; for the sake of simplicity, *M* = 2). None of them directly affected the categorisation mechanisms. The perceptual distances of colour stimuli in the CIE *L^*^a^*^b^*^* space determined the emergent linguistic categories.

To reveal the dynamics of colour categorisation in the model, we conducted additional simulations under different numbers of agents (*N*, 50, 100, and 200). We fixed the number of communications per agent (*C*) as 10^7^ to give agents sufficient communications to develop categorisation patterns and let the whole system to reach a stable state. To trace the dynamics and save the running time, we set the sampling points at 10, 30, 50, 70, 90, 100, 300, …, 10^6^, 3×10^6^, 5×10^6^, 7×10^6^, 9×10^6^ and 10^7^ communications per agent, and calculated three measures at each sampling point: *successful rate* (the proportion of communications whereby the hearer’s guess matched the topic), number of linguistic categories, and number of synonymous colour terms. Under each setting, we conducted 110 runs for comparison with the WCS data and statistical analyses.

As shown in Figure 2 in the main text, the categorisation dynamics of our model was similar under different numbers of agents. This dynamics also resembled that of the category game. Take Figure 2(a) as an example, the dynamics of colour categorisation in our model consisted of the following three phases:

*The increasing phase* (during 0 ~ 5000 communications per agent): Since agents initially had no perceptual category or words, the pressure for discrimination boosted the numbers of perceptual and linguistic categories. Many new words were created randomly and associated respectively to newly-created perceptual categories in agents. This kind of synonymy gradually reached a peak. The “time” (number of communications) to reach the peak and the peak value were correlated with the number of agents in a population; See Figure 2(a), the more agents in a population, the late the time reaching the peak and the higher the peak value. These results indicated that the number of agents in a population could induce a certain degree of variation.

*The dropping phase* (during 5000 ~ 10^4^ communications per agent): After reaching the peak, the synonym level started to dry out, in a similar way to the naming game (*59*). At this phase, new perceptual categories kept emerging, but words within and between agents started to expand their reference across neighbouring perceptual categories in the CIE *L^*^a^*^b^*^* space, thus merging perceptual categories into *linguistic categories*. Linguistic categories also merged in a similar manner, thus causing the total number of linguistic categories to drop. This coarsening of categories followed the similar dynamics to the physical transformation of a glass-forming liquid into a glass under rapid cooling (*60*). The decreasing phase of synonyms was accompanied by the rising phase of successful rate. When on average only one word was recognised by the population for each linguistic category, the synonym level reached to 1.

*The stabilizing phase* (after 10^4^ communications per agent): At this phase, the coarsening of categories became slower and slower, and the linguistic categorisation pattern reached over 85% degree of sharing among agents and remained stable for a plateau phase. The mean numbers of linguistic categories at this state became similar and stable across populations having different numbers of agents. The numbers of emergent linguistic colour categories under the real stimuli (7.526 ± 1.893 under 50 agents at 10^7^ communications per agent; 8.252 ± 2.033 under 100 agents; and 9.445 ± 2.480 under 200 agents) and those under the random stimuli (8.100 ± 2.659; 9.112 ± 2.821; and 10.221 ± 3.892) were all within the range of the number of colour categories as shown in the WCS. This suggested that the stable number of emergent linguistic categories in simulations was less dependent on the number of agents.

Extended Data Figure 1 showed that the dynamics of the model remained similar under different numbers of colour stimuli. In these simulations, to save the running time, we fixed the number of agents as 100, and the number of communications per agent as 10^6^. Based on the 2734 real stimuli, we created five subsets of colour stimuli, having respectively 330, 500, 1000, 1500, and 2000 stimuli randomly chosen from the total 2734 real stimuli. We conducted 110 runs under each set of stimuli. We repeated the steps of stimuli selection and simulation under chosen stimuli for five times, and visualized the dynamics using the same measures (successful rate, number of linguistic categories and number of synonymous colour terms) for each time.

As shown in Extended Data Figures 1(a)‒(e), the model dynamics in all five cases remained similar, consisting of roughly the same three phases as discussed above. In addition, with increase in the number of colour stimuli, the successful rate slightly dropped, and at the stabilizing phase (after 10^4^ communications per agent), the stable number of linguistic categories decreased and the degree of synonymy eventually decreased to 1. These results indicated that the number of colour stimuli could induce a certain degree of variation during the evolution, but such variation could not greatly influence the dynamics of linguistic colour categorisation.

**S1.5. Reasons for not manipulating socio-cultural transmissions**

A complete factorial design based on two factors, namely the perceptual constraints on colours and the socio-cultural transmissions, should manipulate both factors. However, the current study manipulated only the perceptual constraints towards colours. This setting is due to the following reasons.

First, generally speaking, there are three major forms of linguistic cultural transmissions (*61*), including: horizontal transmissions, individuals of the same generation talk to each other; vertical transmissions, individuals of the previous generation talk to their biological offspring in the next generation; and oblique transmissions, individuals of the previous generation talk to non-biological offspring in the next generation. The first form is also referred to as within-generation transmissions, and the latter two as cross-generation transmissions. In reality, all these forms of transmissions are intermingled in a multi-individual, multi-generation community, and they collectively constitute the venue of cultural evolution of language. Previous simulation studies illustrated that: various forms of transmissions play similar roles of information exchange during language evolution (*62-64*); and slightly varying the ratios of these forms of transmissions in total transmissions would not greatly affect mutual understandability of the emergent communal language (*65*). Results could be significantly distinct in some extreme cases. For example, if one individual talks only to his/her offspring and there are no contacts between offspring of different individuals, language would diverge and mutual understanding at the community level would become impossible. Recent experimental semiotics studies also highlight the role of horizontal transmission in maintaining a high level of mutual understanding at the community level (*66*).

Considering that our model simulated how a group of artificial agents developed from scratch a common set of linguistic colour categories through iterated communications, we simulated horizontal transmissions as a representative form of socio-cultural transmission. Given the similar roles of various forms of cultural transmissions in establishing a communal language consisting of common linguistic colour categories, we did not manipulate cultural transmissions by simulating other forms of transmissions, and reasonably expected that the results under other forms of transmissions would be similar (except for extreme cases), as indicated by previous studies (*62-64*). If we want to investigate other issues such as diffusion and/or divergence of linguistic colour categories via communications between social groups, we would need to manipulate cultural transmissions, in line with the settings as in previous studies (*67,68*). We leave this as a promising future work of the current study.

Second, in addition to various forms of cultural transmissions, another factor that could influence language evolution within a group of individuals is social structure; various types or frequencies of connections between individuals in a population could affect the diffusion of linguistic innovations or conventions in the whole population. Based on the network approach, we could transform different types of social structures into different types of networks. Some work based on the category game (*69,70*) or other models (*71,72*) illustrated that popular networks in human communities (e.g., small-world, scale-free, regular, or fully-connected networks) could influence the alignment of common categories across individuals, the level of successful rate, the number of shared linguistic categories, or the diffusion dynamics of common vocabulary. A recent simulation study (*73*) revealed that in large groups, linguistic conventions that are easy to learn (individuals can learn it after a small number of encountering these conventions), such as words, tend to proliferate, whereas small groups in which everyone frequently talks to everyone else allow for more complex conventions, like grammatical regularities, to be maintained. These findings unified the other studies in this line of research. However, none of these studies examined in detail whether the emergent or shared language structures would be significantly distinct under different types of networks. Nonetheless, considering that many languages in the WCS were spoken in small-scale societies and there lacked empirical information about the social structures of these communities, a fully-connected network as simulated in the current study was a reasonable baseline to denote social settings of those communities. Even in this simple, neutral structure, our study demonstrated that different perceptual constraints would induce different linguistic colour categorisation patterns. This illustrated the predominant role of perceptual constraints over social structures in determining the emergent linguistic categorisation pattern. After clarifying the determinant role between perceptual constraints and socio-cultural transmissions in the universality of linguistic colour categorisation, we can move ahead to examine how different social structures affect the dynamics of linguistic categorisation and induce category boundary variations across languages.

Finally, another way of manipulating socio-cultural transmissions is to allow such transmissions or not. For example, some models simulate the transmission of linguistic colour categories via genetic transmission (*21*). However, there lacks evidence of genetic encoding of linguistic colour categories (or other linguistic components); what is possibly encoded in genes is relevant learning abilities that might not be language-specific (*21,44*). In addition, genetic encoding of linguistic components is unable to catch up with the frequent change of environment (*74*), and many models have shown that socio-cultural transmissions together with individual learning abilities achieve similar roles of transmitting linguistic knowledge across generations (*21,65*). Considering these, our model simulates no other means but socio-cultural transmissions.

##### S2. Extended Data Figures and Tables

**
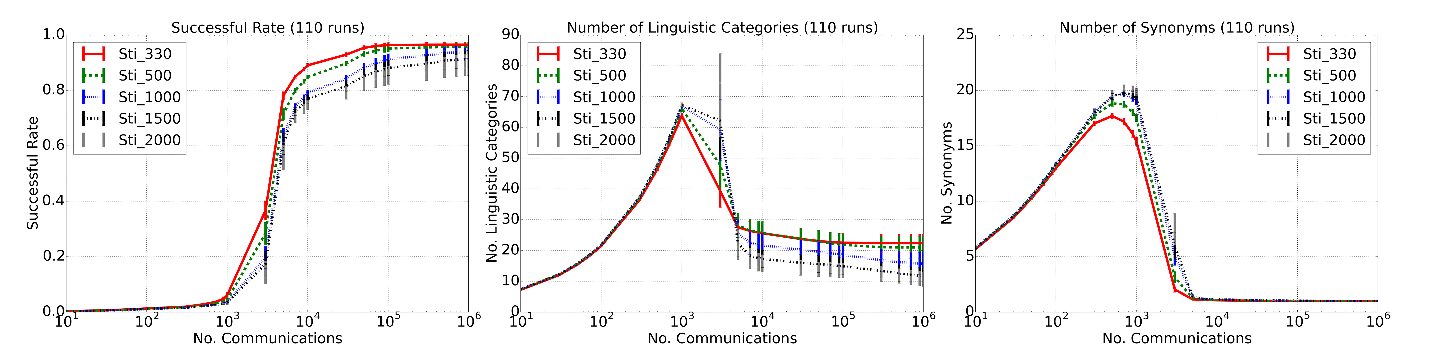
**

**(a)**

**
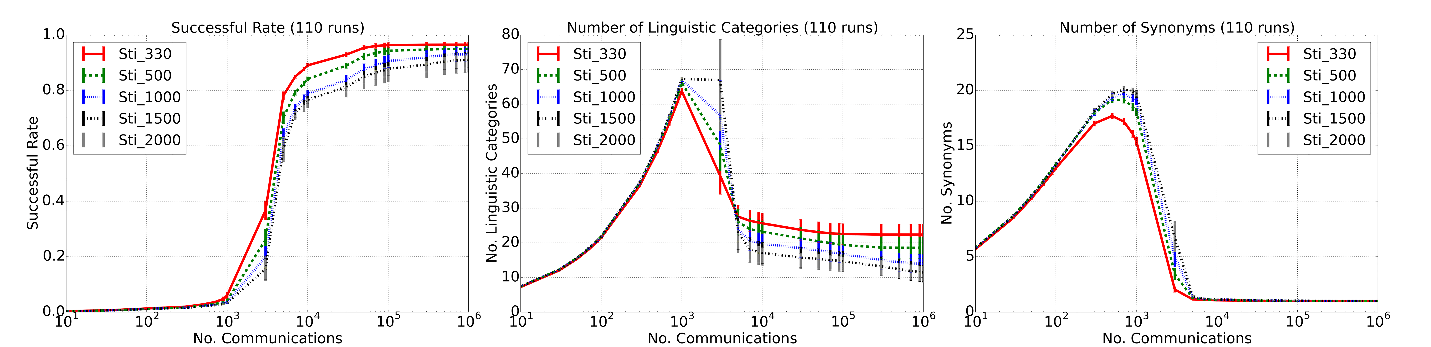
**

**(b)**

**
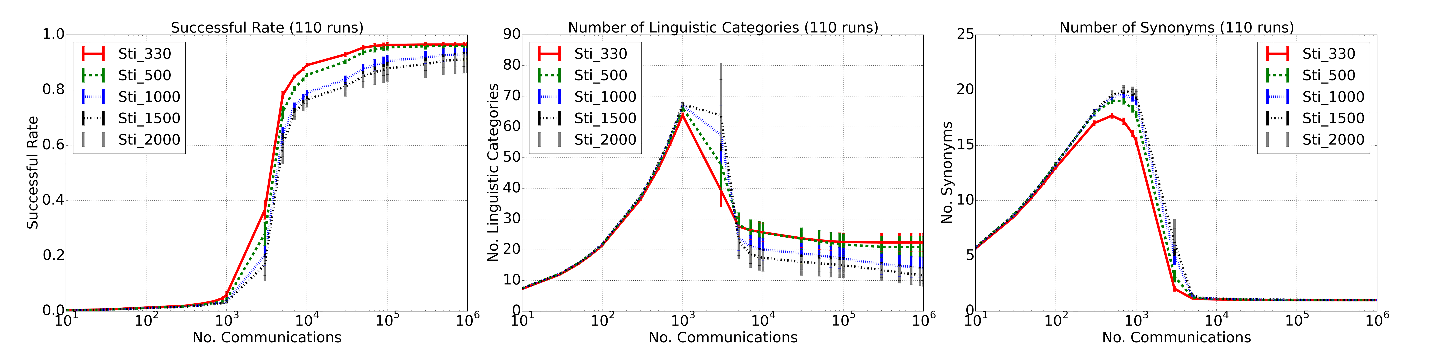
**

**(c)**

**
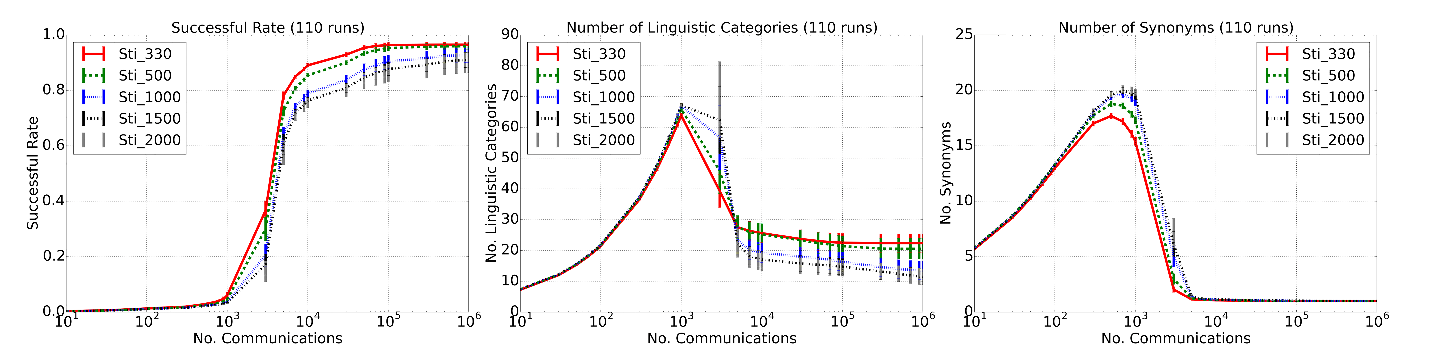
**

**(d)**

**
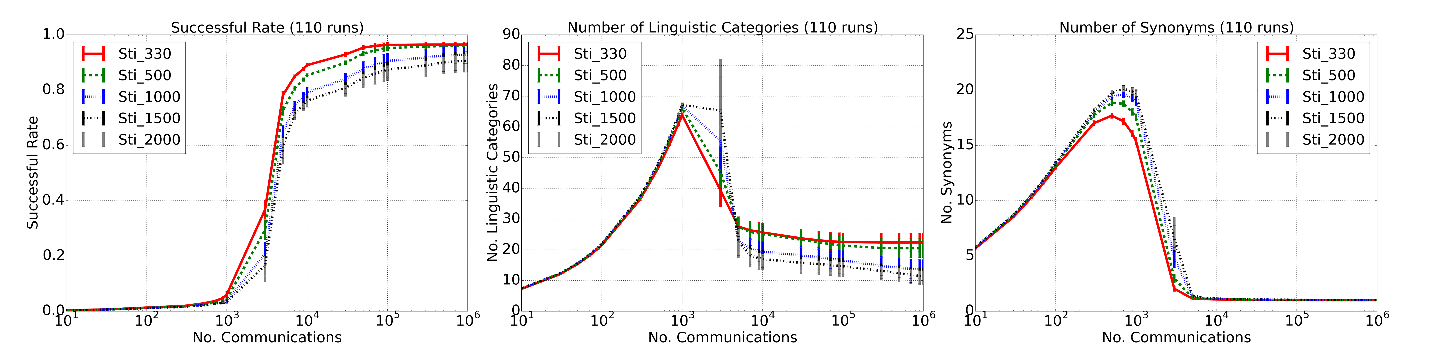
**

**(e)**

**Extended Data Figure 1** Dynamics of the model under different numbers (330, 500, 1000, 1500, and 2000) of colour stimuli. Figures (a) to (e) show the results of five groups of simulations. In each group, there are five sets of simulations, each under a specific number (330, 500, 1000, 1500, or 2000) of colour stimuli randomly chosen from the 2734 real stimuli. In each set, there are 110 runs. The three panels in figures (a) to (e) illustrate respectively the three evaluating indices, including: successful rate, number of linguistic categories, and number of synonyms obtained under specific sets of colour stimuli. Results are averaged over 110 runs in each set. Error bars denote two times of standard errors. To save the running time, all these results are obtained under 100 agents. It is obvious that the dynamics of the model under different numbers of colour stimuli are almost identical, indicating that socio-cultural transmissions play a much similar role in terms of establishing and spreading common colour categories among agents.

**
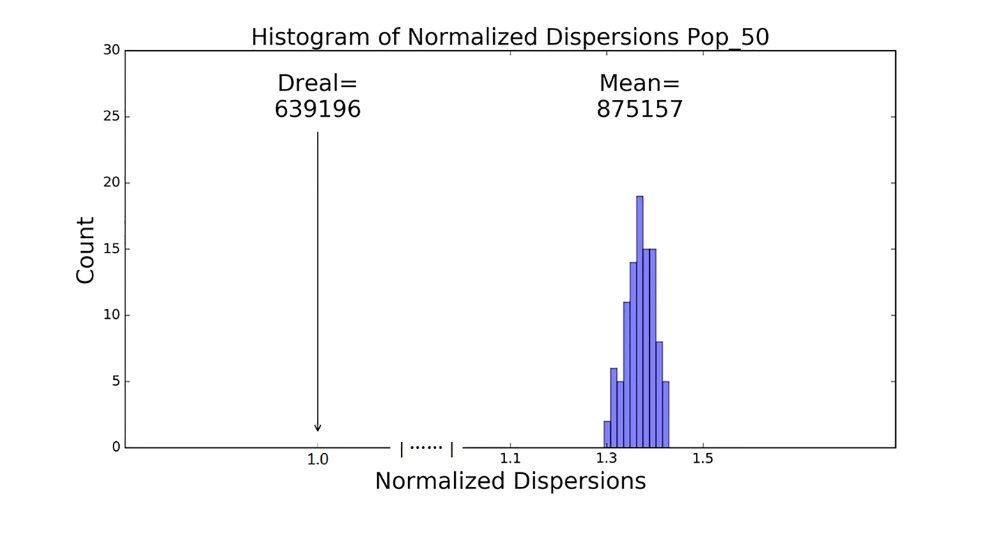
**

**Extended Data Figure 2** Comparison between the cross-language dispersion of the emergent languages obtained under the real stimuli at 10^7^ communications per agent (denoted as $D_{Real}$) and the cross-language dispersions of 100 sets each containing 110 languages obtained under a different set of randomized stimuli (denoted as $D_{Rand}^{''}$). To save the running time, these results are obtained under 50 agents. In the figure, the x-axis is normalized over $D_{Real}$, and the arrow points to $D_{Real}$, and the value after $D_{Rand}^{''}$ is the mean dispersion over the 100 sets of random languages.


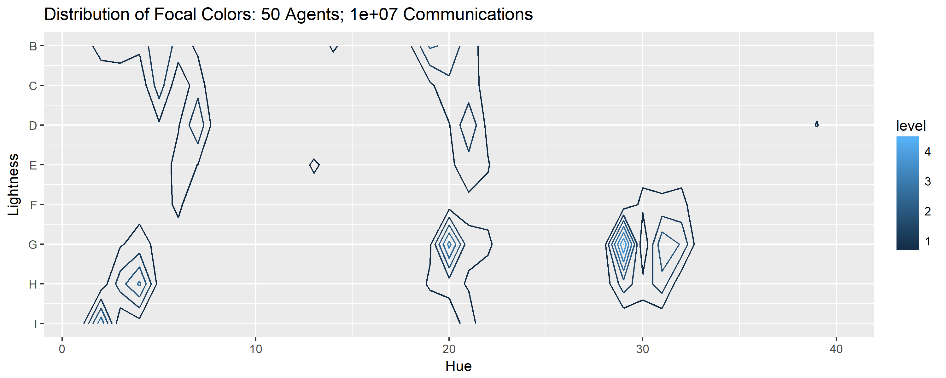


**(a)**


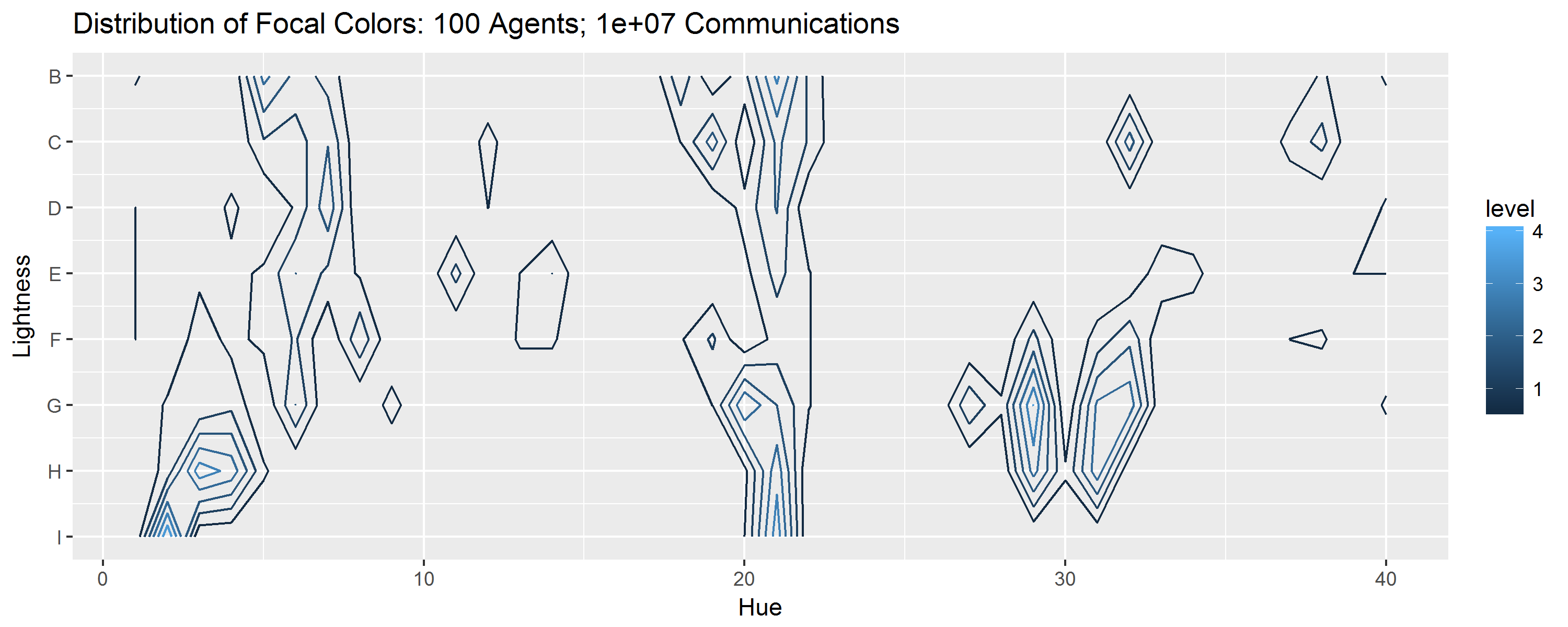


**(b)**

**Extended Data Figure 3** Focal colour distributions at 10^7^ communications per agent under the 2734 real stimuli and 50 agents (a) and 100 agents (b). Results are obtained from 110 runs in each condition. Here, we only show the distribution of emergent focal colours on the 320 colourful stimuli in the Munsell colour stimulus array. The peak-to-peak distance between (a) and Figure 4(c) in the main text is 9.834, and that between (b) and Figure 4(c) is 10.910.

**
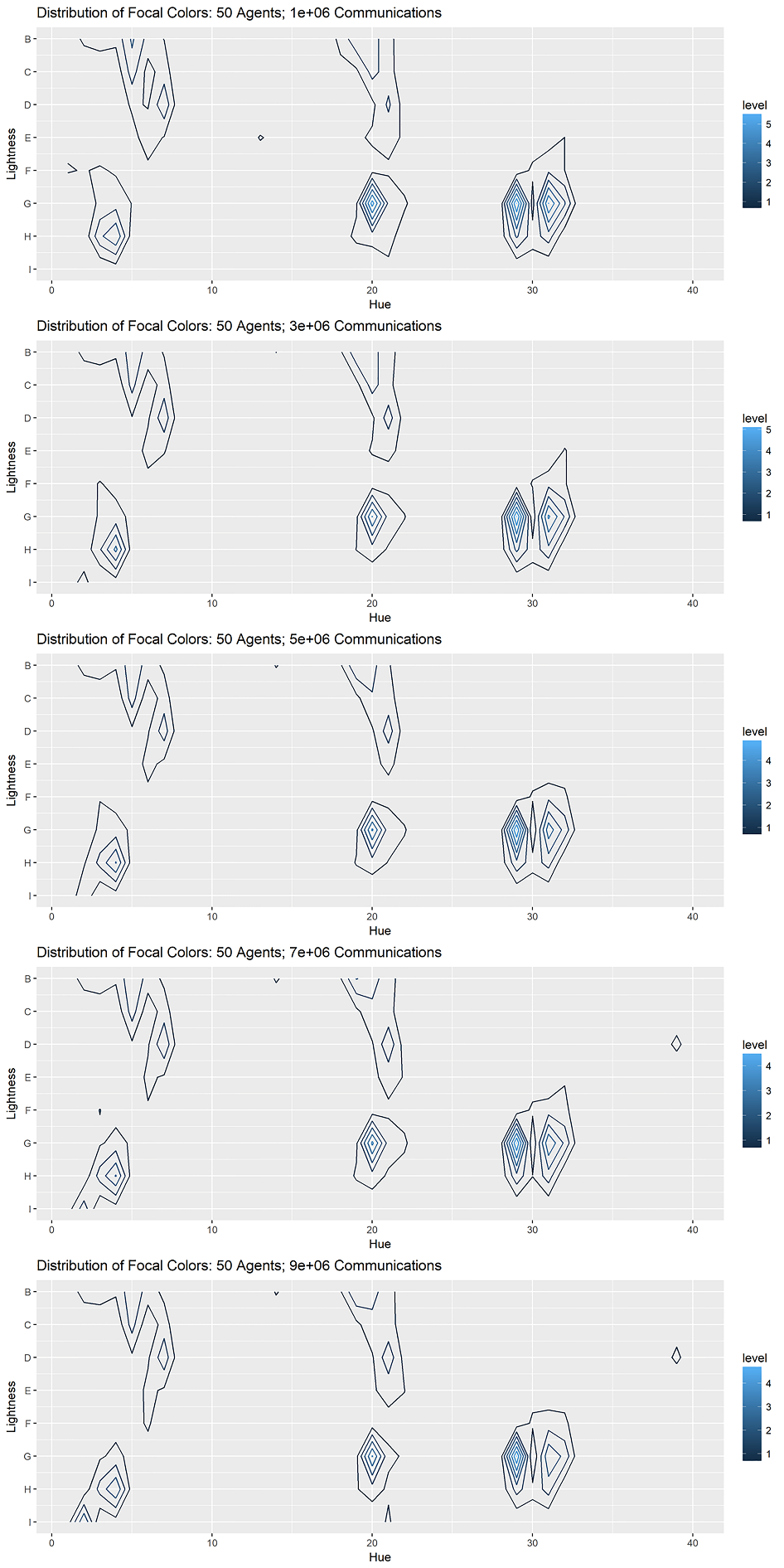
**

**(a)**

**
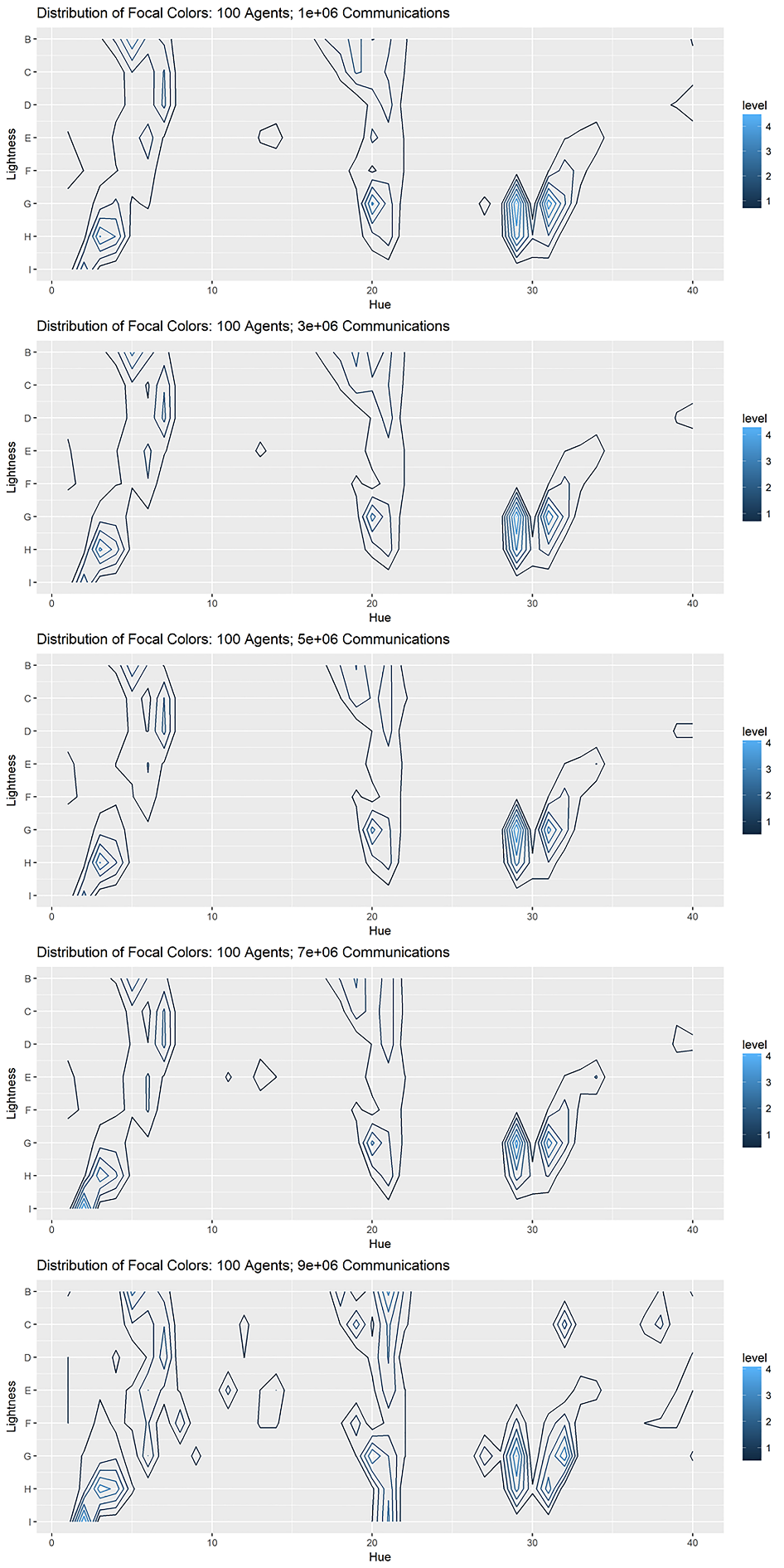
**

**(b)**

**
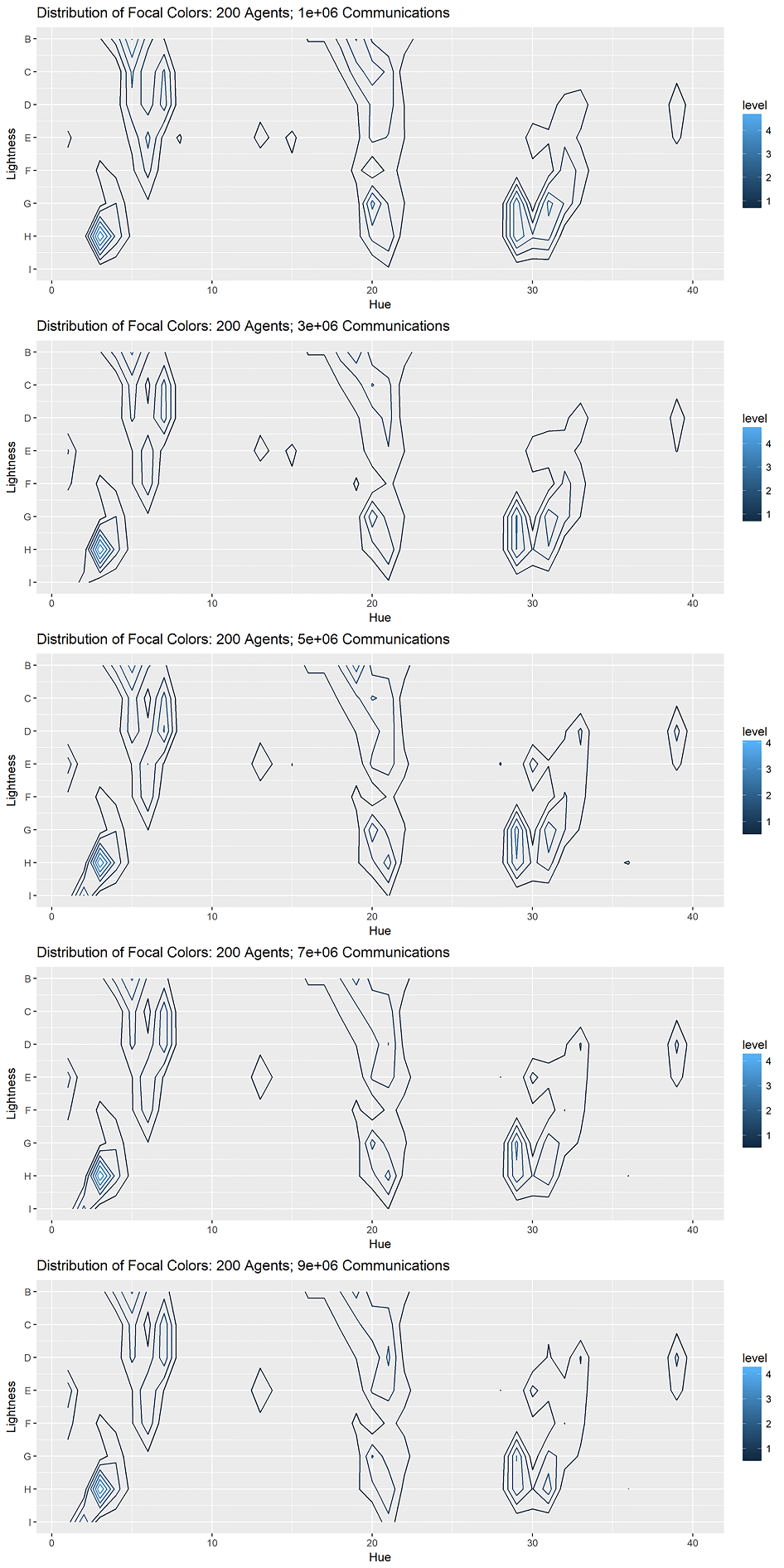
**

**(c)**

**Extended Data Figure 4** Focal colour distributions after 10^6^, 3×10^6^, 5×10^6^, 7×10^6^, 9×10^6^ communications per agent under the 2734 real stimuli and 50 agents (a), 100 agents (b), and 200 agents (c). Results are obtained from 110 runs in each condition. The focal colour distributions have become largely the same once the simulations reach the third stage of the dynamics (see Supplementary Text). Here, we only show the distribution of emergent focal colours on the 320 colourful stimuli in the Munsell colour stimulus array.


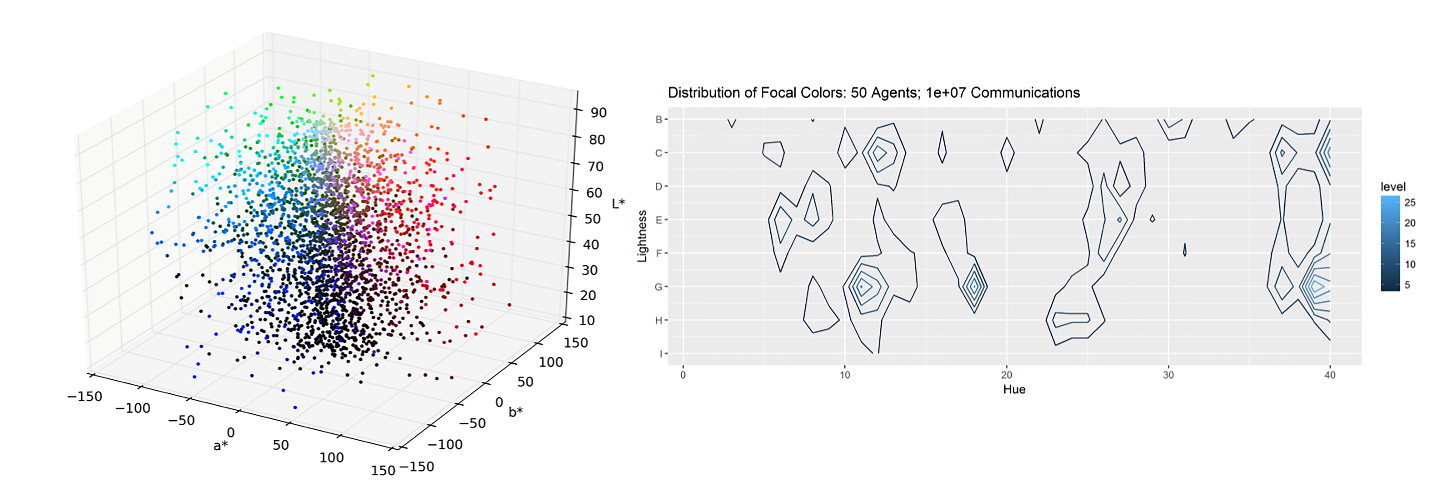


**(a)**

**
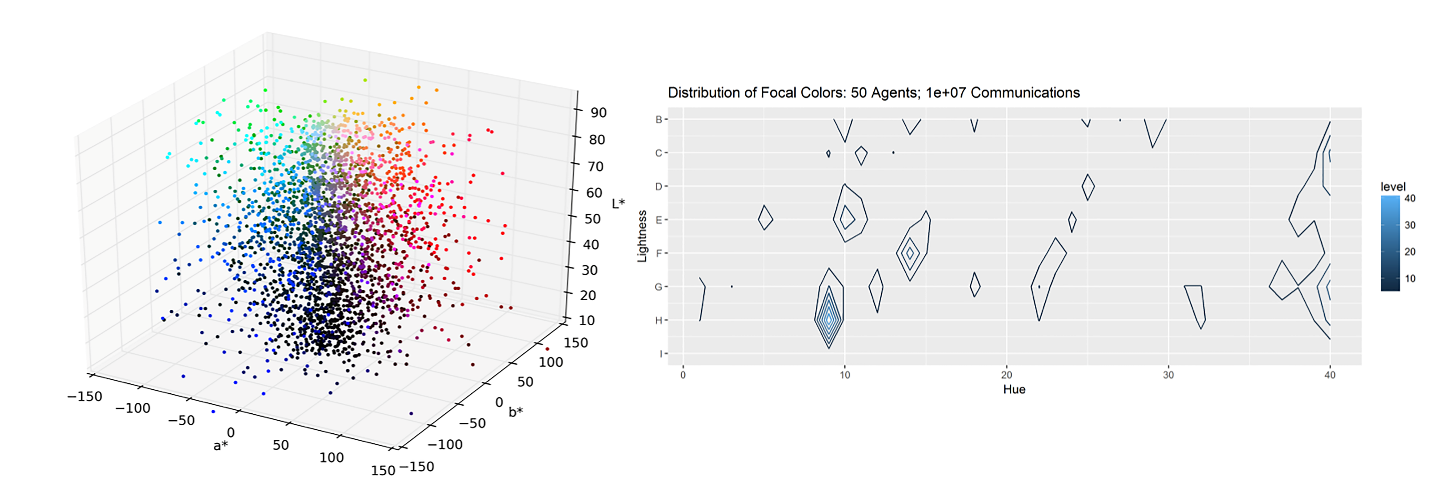
**

**(b)**

**
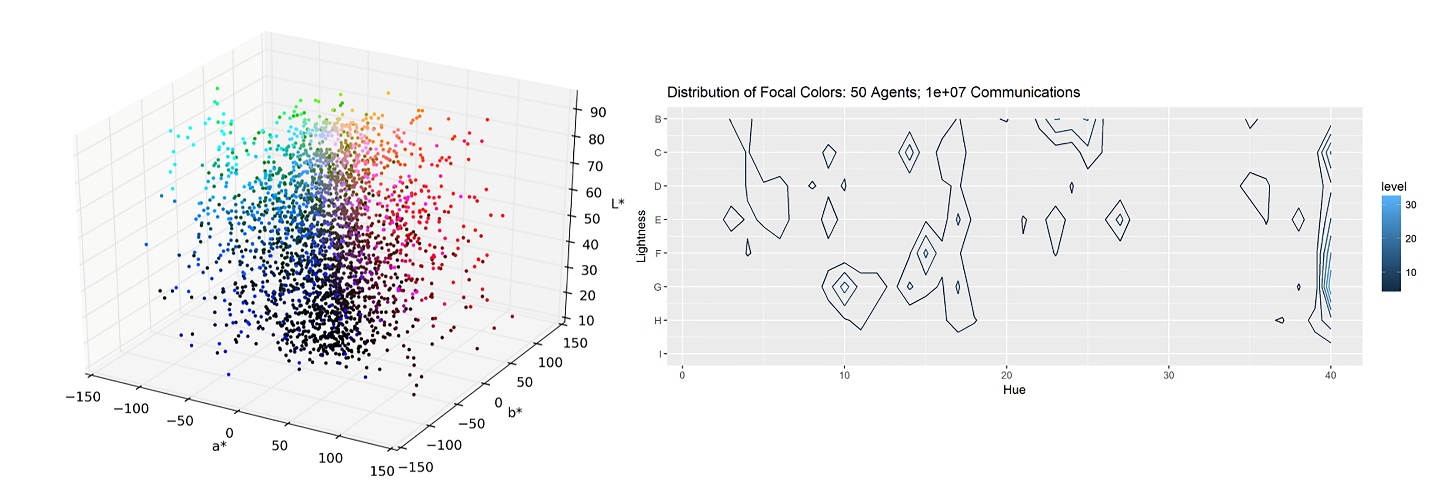
**

**(c)**

**
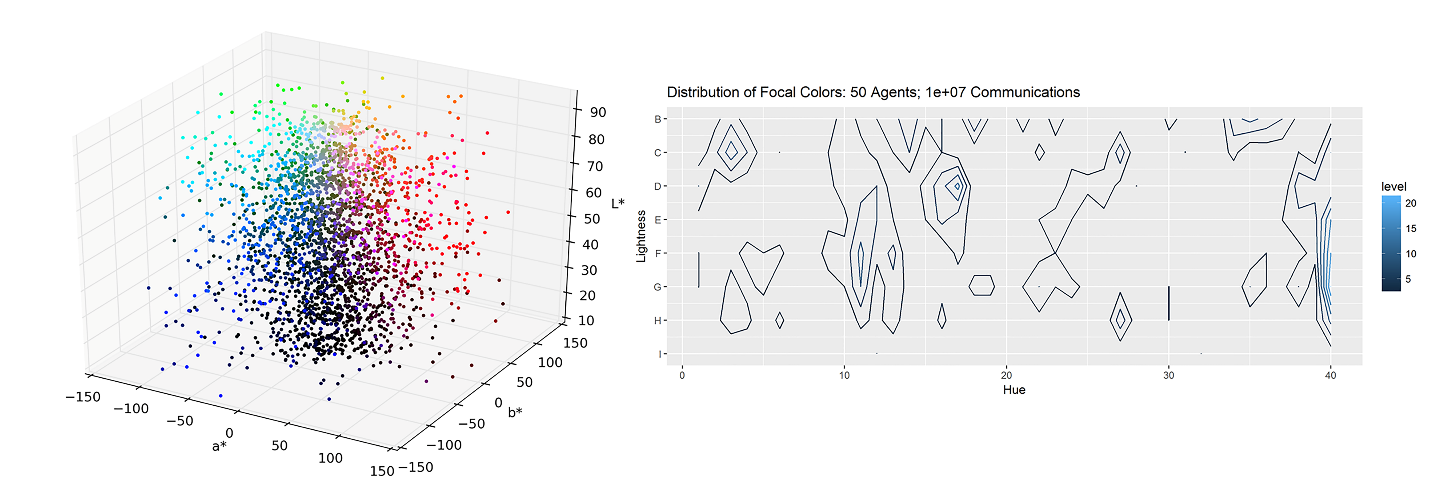
**

**(d)**

**
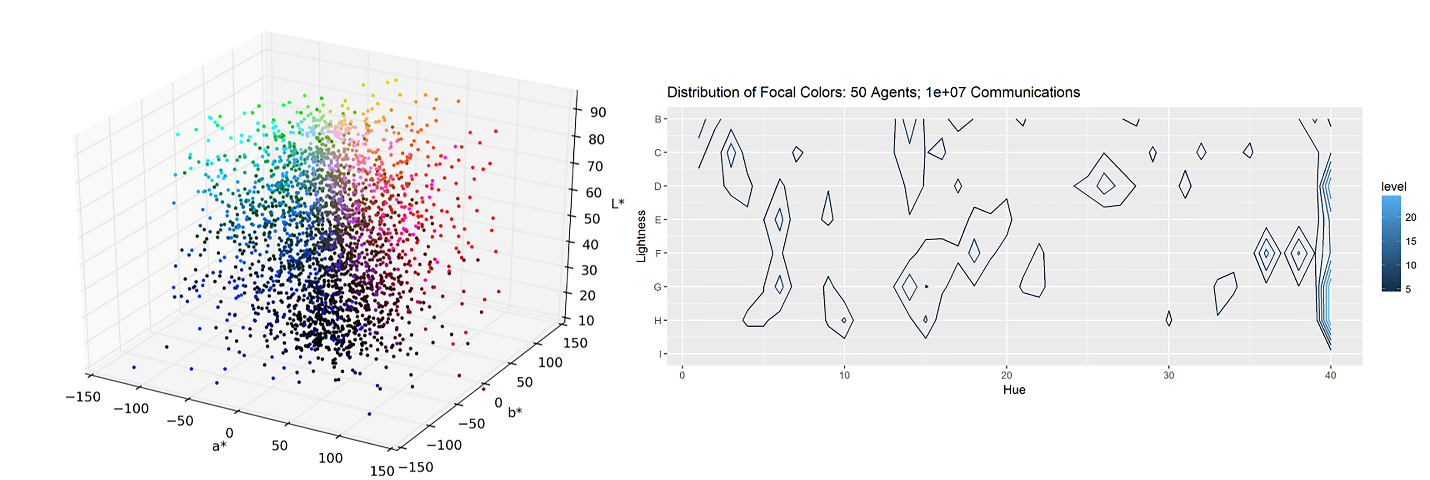
**

**(e)**

**Extended Data Figure 5** Focal colour distributions under another five sets of randomized stimuli. Figures (a) to (e) show the results of five sets of simulations, each under a different set of randomized stimuli. In each figure, the left panel shows the distribution of the randomized stimuli in the CIE *L^*^a^*^b^*^* space, and the right panel shows the focal colour distribution obtained after 10^7^ communications per agent. Results are obtained from 110 runs in each condition. To save the running time, results are obtained under 50 agents. The results under other numbers of agents are similar (based on the Extended Data Figures 2). Here, we only show the distribution of emergent focal colours on the 320 colourful stimuli in the Munsell colour stimulus array.

**Extended Data Table 1.** Peak-to-peak distances between the emergent focal colours under the real stimuli and the focal colours in the WCS languages.

| **No. Agents \ Communications per agent** | **10^6^** | **3×10^6^** | **5×10^6^** | **7×10^6^** | **9×10^6^** | **10^7^** |
| --- | --- | --- | --- | --- | --- | --- |
| **50** | 15.089 | 12.060 | 9.834 | 9.834 | 9.834 | 9.834 |
| **100** | 16.958 | 13.716 | 11.239 | 11.239 | 10.910 | 10.910 |
| **200** | 18.842 | 16.958 | 15.716 | 15.662 | 14.628 | 14.628 |

**Extended Data Table 2.** Peak-to-peak distances between the emergent focal colours under the randomized stimuli and the focal colours in the WCS languages.

| **No. Agents \ Communications per agent** | **10^6^** | **3×10^6^** | **5×10^6^** | **7×10^6^** | **9×10^6^** | **10^7^** |
| --- | --- | --- | --- | --- | --- | --- |
| **50** | 18.728 | 18.750 | 17.711 | 17.873 | 17.873 | 17.873 |
| **100** | 25.504 | 20.227 | 20.337 | 20.501 | 21.079 | 21.079 |
| **200** | 28.923 | 26.612 | 27.793 | 24.333 | 24.888 | 21.475 |

**Extended Data Table 3.** Peak-to-peak distances between the emergent focal colours under the real stimuli and those under the randomized stimuli.

| **No. Agents \ Communications per agent** | **10^6^** | **3×10^6^** | **5×10^6^** | **7×10^6^** | **9×10^6^** | **10^7^** |
| --- | --- | --- | --- | --- | --- | --- |
| **50** | 16.769 | 18.500 | 18.448 | 18.448 | 18.448 | 18.448 |
| **100** | 19.811 | 15.394 | 13.728 | 14.875 | 17.323 | 21.919 |
| **200** | 22.008 | 25.106 | 19.586 | 20.859 | 22.644 | 20.322 |

**S3. Additional References**

1. Kay, P. & Regier, T. Colour naming universals: The case of Berinmo. *Cognition* **102(2)**, 289-298 (2007).
2. CIE. *A Colour Appearance Model for Colour management systems: CIECAM02* (CIE Publ. 159, 2004).
3. Berns, R. S. *Billmeyer and Saltzman’s Principles of Colour Technology*. 3rd edition. John Wiley & Sons. (2000).
4. Li, D., Fan, Z. & Tang, W. K. S. Domain learning naming game for colour categorisation. *PLOS ONE* **12**, e0188164 (2017).
5. Medin, D. L. & Schaffer, M. M. Context theory of classification learning. *Psycho. Rev.* **85**, 207-238 (1978).
6. Smith, E. & Medin, D. The exemplar view. In E. Margolis & S. Laurence (Eds.), *Concepts: Core readings* (pp. 207-209). Cambridge, MA: MIT Press (1999).
7. Reisberg, D. *Cognition: Exploring the Science of the Mind*. 5th edition. New York: W. W. Norton & Co. (2013).
8. Lakoff, G. *Women, Fire, and Dangerous Things*. Chicago, IL: University of Chicago Press (1987).
9. Rosch, E. H. Natural categories. *Cog. Psycho.* **4**, 328-350 (1973).
10. Rosch, E., H. Cognitive representations of semantic categories. *J. Exp. Psycho.: General* **104(3)**, 192-233 (1975).
11. Smith, J. D. & Minda, J. P. Thirty categorisation results in search of a model. *J. Exp. Psycho.: Learn., Mem., & Cog.* **26**, 3-27 (2000).
12. Storms, G., De Boeck, P. & Ruts, W. Prototype and exemplar-based information in natural language categories. *J. Mem. Lang.* **42**, 51-73 (2000).
13. Feldman, J. The simplicity principle in human concept learning. *Curr. Dir. Psycho. Sci.* **12**, 227-232 (2003).
14. Baronchelli, A., Felici, M., Loreto, V., Caglioti, E. & Steels, L. Sharp transition towards shared vocabularies in multi-agent systems. *J. Stat. Mech.: Theory & Experiment*, P06014 (2006).
15. Fisher, M. E. & Barber, M. N. Scaling theory for finite-size effects in the critical region. *Phys. Rev. Lett.* **28(23)**, 1516-1519 (1972).
16. Mesoudi, A. & Whiten, A. The multiple roles of cultural transmission experiments in understanding human cultural evolution. *Philoso. Trans. Roy. Sco. B.* **363**, 3489-3501 (2008).
17. Smith, K., Brighton, H. & Kirby, S. Complex systems in language evolution: The cultural emergence of composition structure. *Adv. Comp. Sys.* **6(4)**, 537-558 (2003).
18. Vogt, P. & Lieven, E. [Verifying theories of language acquisition using computer models of language evolution](http://ilk.uvt.nl/~paul/publications/abVogtLieven2010.pdf). *Adapt. Behav.* **18(1)**, 21-35 (2010).
19. Acerbi, A. & Parisi, D. Cultural transmission between and within generations. *J. Arti. Soc. Soc. Sys.* **9** (2006)
20. Gong, T. Exploring the roles of horizontal, vertical, and oblique transmissions in language evolution. *Adapt. Behav.* **18(3-4)**, 356-376.
21. Tamariz, M. & Kirby, S. [The cultural evolution of language](https://www.sciencedirect.com/science/article/pii/S2352250X15002225). *Curr. Opin. Psychol.* **8**, 37-43 (2016).
22. Axelrod, R. The dissemination of culture: A model with local convergence and global polarization. *J. Confl. Resol.* **41(2)**, 203-226 (1997).
23. Gong, T., Shuai, L. & Ansaldo, U. Computer simulation of language convergence. In: Peng, G. & Shi, F. (Eds.), *Eastward Flows the Great River: Festschrift in Honor of Prof. William S-Y. Wang on His 80th Birthday* (pp. 375–392). Tianjin, China: Nankai University Press (2013).
24. Gong, T., Puglisi, A., Loreto, V. & Wang, W. S.-Y. Conventionalization of linguistic categories under communicative constraints. *Biological Theory* **3(2)**, 154–163 (2008).
25. Gong, T., Baronchelli, A., Puglisi, A. & Loreto, V. Exploring the roles of complex networks in linguistic categorisation. *Artificial Life* **18**, 107-121 (2012).
26. DallʼAsta, L., Baronchelli, A., Barrat, A. & Loreto, V. Nonequilibrium dynamics of language games on complex networks. *Phys. Rev. E* **74(3)**, 036105 (2006).
27. Gong, T., Minett, J. W. & Wang, W. S-Y. Exploring social structure effect on language evolution based on a computational model. *Connection Science* **20(2-3)**, 135-153 (2008)
28. Reali, F., Chater, N. & Christiansen, M. H. Simple grammar, large vocabulary: How population size affects language. *Proc. R. Soc. B.*, **285**, 20172586 (2018).
29. Christiansen, M. H. & Chater, N. Language as shaped by the brain. *Behav. Brain Sci.* **31**, 489-558 (2008).
